# Copper-Catalyzed Dual Cyclization for the Synthesis of Quinindolines

**DOI:** 10.3390/molecules25225303

**Published:** 2020-11-13

**Authors:** Hung-Kai Wang, Yu-Lun Chio, Gangaram Pallikonda, Hsyueh-Liang Wu, Haw-Lih Su, Jen-Chieh Hsieh

**Affiliations:** 1 Department of Chemistry, Tamkang University, New Taipei City 25137, Taiwan; zzz759153@gmail.com (H.-K.W.); b45678955@gmail.com (Y.-L.C.); gangaram.palli@gmail.com (G.P.); 2 Department of Chemistry, National Taiwan Normal University, Taipei 11677, Taiwan; 3 Central Laboratories Unit, Qatar University, Doha 999043, Qatar; hawlih@gmail.com

**Keywords:** copper-catalyzed, quinindoline, natural alkaloid, *N*-heterocycle, cyclization

## Abstract

A synthetic approach to quinindoline derivatives by the Cu-catalyzed dual cyclization has been developed. This catalytic reaction is a practical method for the systematic synthesis of quinindoline core structure, which contains a limited-step synthetic strategy and can tolerant a wide variety of substituents. In addition, the mechanistic study reveals that the reaction initiates from a Lewis acid accelerated addition of aniline to nitrile and provides the indole substructure, and then the subsequent Cu-catalyzed C-N coupling reaction furnishes the quinoline subunit and affords the quinindoline structure.

## 1. Introduction

**Figure 1:**
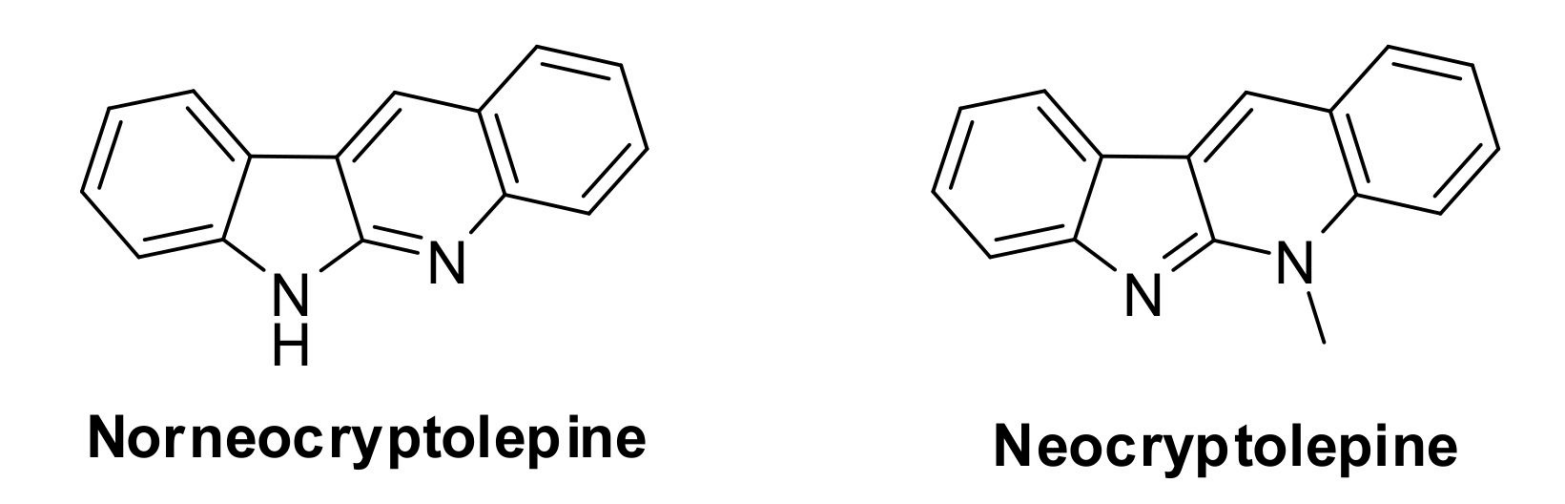
Natural alkaloids with quinindoline core structure.

Quinindoline, the tetra-fused *N*-heterocycle, is an important structure, particularly in the field of medical and biological chemistry, which contains the common bioactive subunits quinoline and indole in its skeleton. The most useful and representative natural alkaloids containing the simplest substituent on the quinindoline core structure are norneocryptolepine (without any substituent on the core) and neocryptolepine (with a solely *N*-methyl group) [1,2,3] (Figure 1). These two important alkaloids have attracted considerable attention from chemists because of their versatile bioactivities, including antimalaria, antitumor, antibacterial properties [4,5,6]. They are the important components of traditional herbal medicine in the West and Central Africa [7], and are also the model structures for the design of pharmaceutical compounds. Therefore, many related studies, for example, their structure–activity relationship, strategies for the synthetic approaches, and spectroscopy have been developed in recent decades [8].

**Scheme 1:**
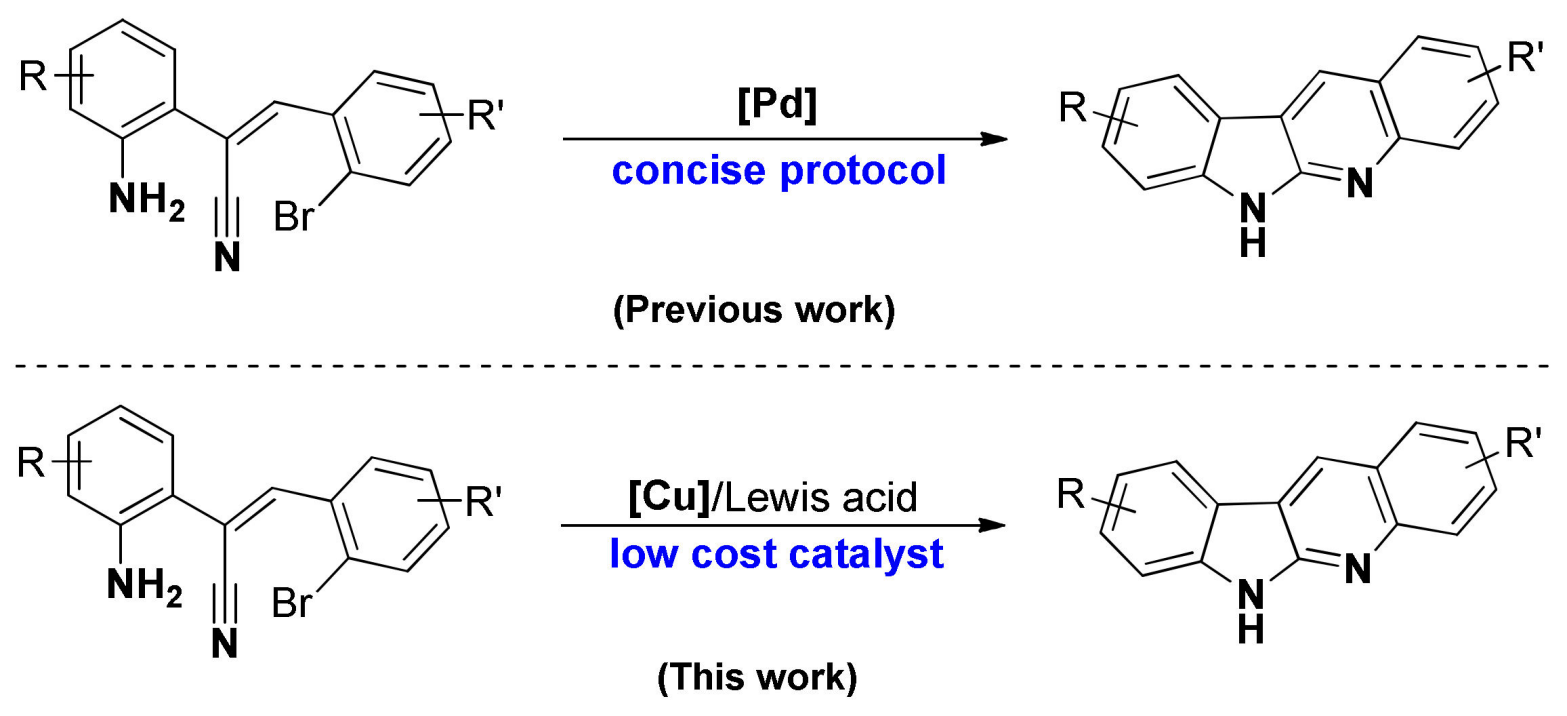
Palladium and copper-catalyzed dual cyclization to synthesize quinindolines.

Because of the complicity in the structure of tetra-fused rings, the developed methods for the synthesis of quinindolines cannot satisfy the comprehensiveness of synthesis on the structures, and thus so many different approaches appeared based on this. The typical approaching routes mainly relied on three pathways. The first one is extension of two aromatic rings from an indole substrate, which is also the most commonly involved strategy [9,10,11,12,13]. The second route is the ring expansion of a quinoline derivative. This is the oldest strategy to afford the quinindolines, and reported as the first method to synthesize the neocryptolepine [14,15,16]. The dual annulation of an *N*-alkynylaryl-*N*-aryl carbodiimide through diradical pathway is the third common strategy, but not as often recorded as other two routes [17,18,19]. Despite these three dominant pathways, there have been several modern strategies reported [11,12,13,20,21,22]. In this respect, our recent work in the synthesis of norneocryptolepine derivatives through a Pd-catalysis can systematically offer the unprotected quinindolines various substituents on the benzene moieties (Scheme 1) [22]. This work and our experience in the construction of heterocycles [23,24,25,26,27,28,29,30] encouraged us to continuously study in this field. In addition, during the investigation of optimized conditions, we found the possibility to proceed with the dual cyclization under a Cu/Lewis acid catalytic system. This discovery offered an alternative protocol in a lower cost catalytic system and also the potential for industrial applications. Herein, we report a Cu/Lewis acid catalyzed dual cyclization for the synthesis of quinindolines.

## 2. Results and Discussion

Our study was initiated from the inadvertently discovered condition of copper catalysis, although it did not show satisfactory performance (Table 1, entry 1). We then investigated various factors of the conditions for this copper catalysis, and the results are summarized in Table 1 (also see Supplementary Materials in detail). We first examined different solvents and found that because the high reaction temperature is required, only the polar solvents with high boiling points can advance the reaction effectively (entries 2, 3). The cosolvent system is helpful for this reaction (entries 4–6), and the best yield was observed in a 9:1 ratio of DMF/NMP. Increase or decrease in the temperature reduced the reaction yields (entries 7, 8). We also test different kinds of ligand; however, they did not promote the reaction yields at all. When we introduced the Lewis acid BF_3_·OEt_2_ into the reaction, the reaction yield was significantly improved (entry 9). We further reduced the loading of Cu_2_O and increase the amount of BF_3_·OEt_2_, and found that the yield was further increased under 36 h reaction time (entry 10). Other copper sources cannot perform as good as the Cu_2_O (entry 11). The Lewis acid is crucial, and we found that only the boron acids can improve the reaction yield, other Lewis acids such as AlCl_3_, TiCl_4_ and FeCl_3_ are not able to provide the desired product in good yield (entries 12–14). Moreover, in the absence of copper source, the reaction can still proceed in a bad yield (entry 15). We then tried to reduce the loading amount of copper sources by fine-tuning the combination of every factor, but did not well succeed (see Supplementary Materials for the detail). The moderate or better reaction yields required at least 20 mol% of copper sources (10 mol% Cu_2_O).

After studying the factors of the reaction conditions, we then investigated the reaction scope by testing various substituents to understand the capacity of this copper catalysis. We selected two conditions (entries 9, 10) as our standard conditions since we found that different structures fit different conditions. The reactivity for the substituent on the aniline moiety (left ring, Table 2) was surveyed first. It was found that an electron-donating group on the aniline moiety can generally provide higher yields of the desired products than those with an electron-withdrawing group on it. In addition, for the substrates with an electron-donating group, the condition A (10 mol% of Cu_2_O and 40 mol% of BF_3_·OEt_2_) can provide higher yields of the desired products than the condition B (20 mol% of Cu_2_O and 20 mol% of BF_3_·OEt_2_). On the contrary to the electron-donating substituents, the substrates with an electron-withdrawing group can offer higher yields under the condition B. Thus, for a substituent para to the amino group (entries 1–3), the desired product **2b** can be afforded in 41% yield under condition B; and the products **2c**, **2d** and **2e** can be obtained in 68%, 76% and 45% yields, respectively, by using condition A. The substrates with a substituent meta to the amino group (**1f** and **1g**) can also advance the reaction smoothly and afford the corresponding products **2f** and **2g** in moderate yields with condition B. The disubstituted products **2h** and **2i** can be obtained in 71% and 56% yields, respectively, with condition A. 

We further surveyed the substrates with a substituent on the ortho-bromostyryl moiety. As indicated in Table 3, the products were consistently obtained in higher yields with condition A. For the substrates with a substituent para to the bromide (**1j**, **1k** and **1l**), the corresponding products **2j**, **2k** and **2l** can be afforded in moderate-to-good yields. The distribution of yields reveals that the electronic property on the styryl moiety also significantly affects the reaction yields. Substrates with a substituent meta to the bromide advance the reaction smoothly as well. Thus, the meta-fluoro and chloro groups resulted in similar yields of the desired products (**2m** and **2n**), while the meta-methyl substituted product **2o** was generated in a slightly lower yield. The amino substituted products **2p** and **2q** were obtained in much lower yields comparing with other meta-substituted products. The disubstituted products **2r** and **2s** can be also generated in moderate yields; the yields of the desired products are 62% and 57%, respectively. Moreover, the naphthyl group is able to be tolerated to give the product **2t** in 49% yield. 

**Scheme 2:**
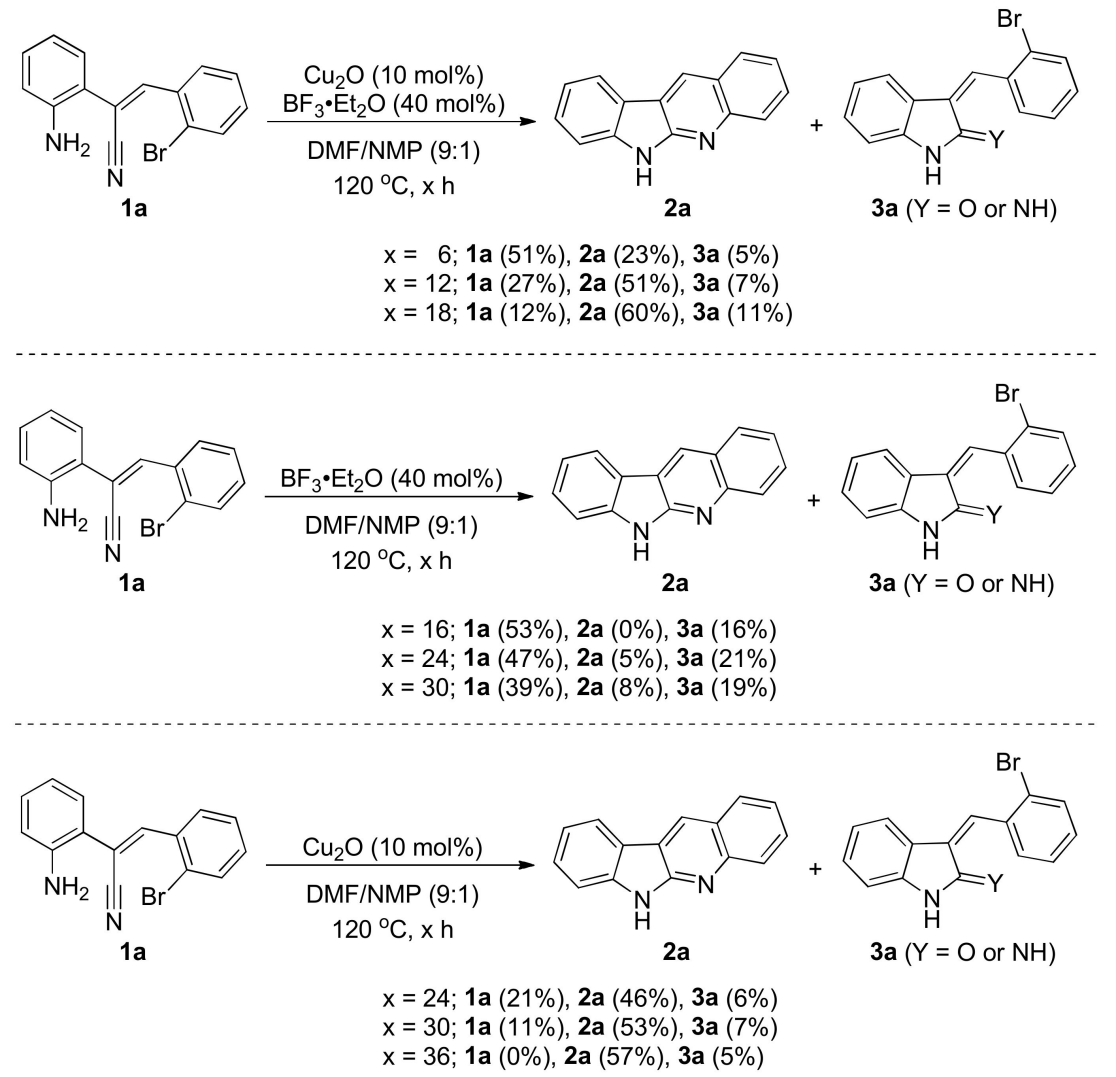
Control experiments ^a^; ^a^ the yields were estimated by GC-MS.

From the results of this and our previous works, we can find that the reactivity of substrates is very different between conditions of the Pd- and the Cu-catalysis. Therefore, we tried to figure out the reaction pathway of this Cu-catalytic dual cyclization. We set up some control experiments to carefully study the nature of this reaction (Scheme 2). We first monitored the relationship between the reaction time and the reaction behavior. When the reaction time was increased, the recovered amount of substrate **1a** decreased, and the yield of product **2a** increased. A supposed intermediate **3a** was detected in low yields. We then removed the copper source and checked the difference of the reaction, and the results were unexpected. The supposed intermediate **3a** was always detected, and the detected amount of **3a** was steady around 20% for the reaction time greater than 16 h. In addition, a trace amount of the desired product **2a** was detected, which was probably caused by the intramolecular S_N_Ar reaction. We further investigated the variety of the amount of **3a** by solely using Cu_2_O as the catalyst. It was found that the substrate is fully consumed in a much longer reaction time compared with the standard condition, and the crude ^1^H NMR spectrum is messier. Some unidentified compounds appear at 18 h reaction time. The amount of supposed intermediate **3a** was generally kept in a low yield for different reaction times. The formation of **3a** implied that the cyclization pathway is likely similar to the reaction with the condition of BF_3_·OEt_2_ only. The results of control experiments can be briefly concluded as the following. First, the supposed intermediate **3a** was detected in every control experiment; therefore, it is highly possible that **3a** is the key intermediate in the copper catalysis. Second, the amount of **3a** is higher while the BF_3_·OEt_2_ is introduced into the reaction. This phenomena is obvious when the BF_3_·OEt_2_ is solely introduced without any copper source. Third, during the reaction, the amount of **3a** is steady, and would not change as the product formed. This is probably because the subsequent step is faster than the formation of **3a**.

**Scheme 3:**
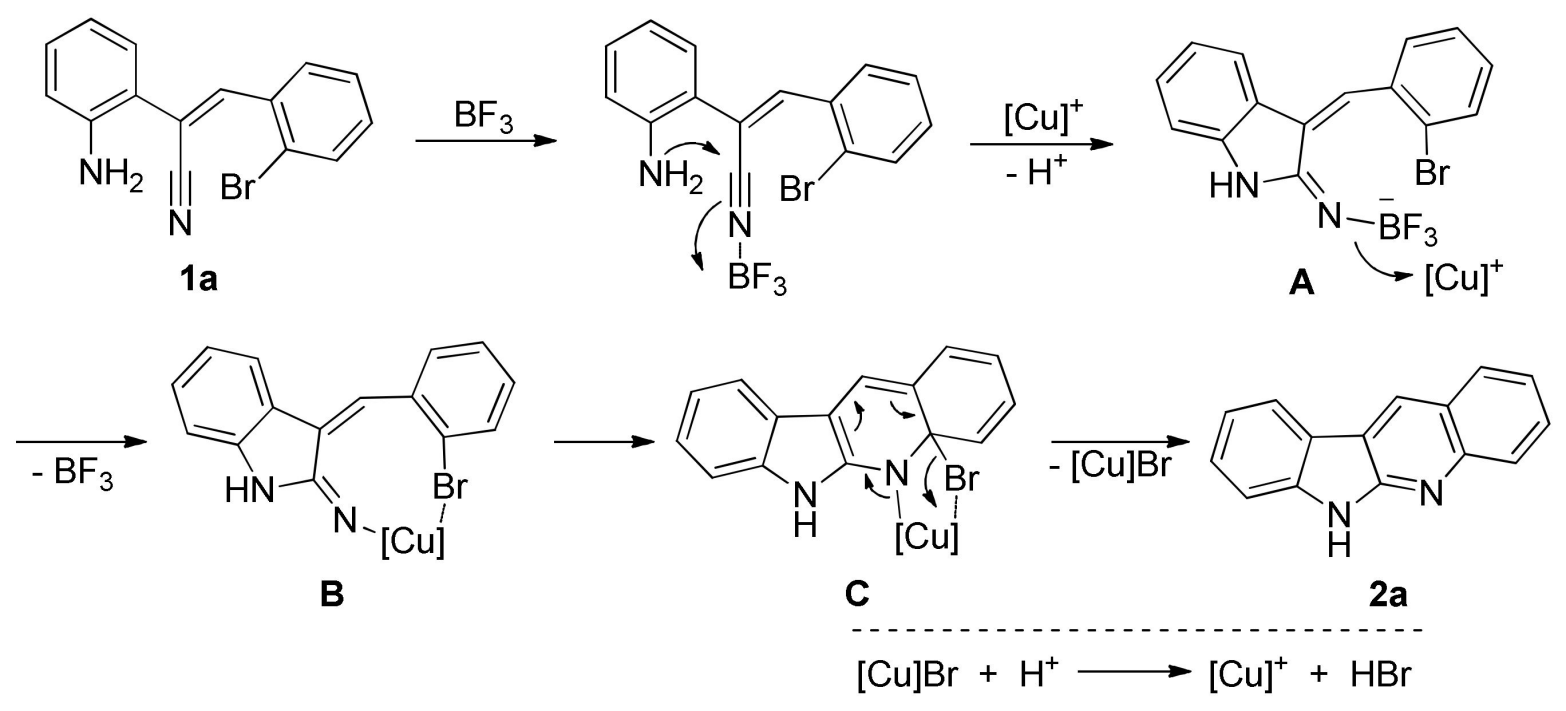
Proposed reaction mechanism.

Based on the above results and the previous report [31], a tentative reaction pathway can be proposed as below (Scheme 3). The reaction is likely to be initiated by the coordination of substrate with BF_3_, which accelerates the cyclization to form the intermediate **A**. Transmetalation of the boron species **A** generates the copper complex **B**, which facilitates the intramolecular addition to form the complex **C**. Aromatization and release of [Cu]Br affords the desired product **2a**. The [Cu]Br can react with proton to regenerate the [Cu]^+^.

## 3. Materials and Methods

All reagents were purchased from Sigma-Aldrich (St. Louis, MO, USA), Alfa-Aesar (Haverhill, MA, USA), TCI (Tokyo, Japan) and Fisher-Acros (Loughborough, UK), and were used without further purification unless otherwise noted. All manipulations of oxygen- and moisture-sensitive materials were conducted with a standard Schlenk technique or in the glove box. Flash column chromatography was performed using silica gel (230–400 mesh). Analytical thin layer chromatography (TLC) was performed on 60 F_254_ (0.25 mm) plates and visualization was accomplished with UV light (254 and 354 nm) and/or an aqueous alkaline KMnO_4_ solution followed by heating. Proton and carbon nuclear magnetic resonance spectra (^1^H NMR and ^13^C NMR) were recorded on Bruker 300 or Bruker 600 spectrometer with Me_4_Si or solvent resonance as the internal standard (^1^H NMR, Me_4_Si at 0 ppm, CDCl_3_ at 7.26 ppm, *d*_6_-DMSO at 2.49 ppm; ^13^C NMR, Me_4_Si at 0 ppm, CDCl_3_ at 77.0 ppm, *d*_6_-DMSO at 39.7 ppm). ^1^H NMR data are reported as follows: chemical shift, multiplicity (s = singlet, d = doublet, t = triplet, q = quartet, quint = quintet, sext = sextet, sept = septet, br = broad, m = multiplet), coupling constants (Hz), and integration. IR spectral data were recorded on a Bruker TENSOR 37 spectrometer (Bruker, Billerica, MA, USA). Melting points (mp) were determined using a SRS OptiMelt MPA100 (Stanford Research Systems, Sunnyvale, CA, USA). GC-MS data were obtained from the HP 5890 Series II GC/HP 5972 GC MASS Spectrometer System. High resolution mass spectral data were obtained from MAT-95XL HRMS by using EI method.

## 4. Conclusions

In conclusion, we have developed a catalytic dual cyclization to approach the quinindolines by using the copper and boron species as the catalytic system. This catalytic cyclization could proceed for most substrates and provide the desired products in moderate-to-good yields with tolerance of various substituents. Study of the reaction mechanism via the control experiments indicates that the reaction goes through the formation of an oxindole-related intermediate, and the copper species can facilitate the intramolecular S_N_Ar reaction. Moreover, this reaction protocol with low cost catalysts may represent a practical synthesis with potential in industrial applications.

## Figures and Tables

**Table 1:** Optimization of reaction conditions ^a^.

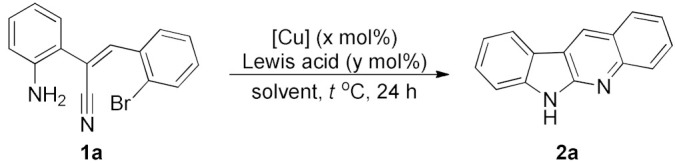					
Entry	[Cu] (x)	Lewis Acid (y)	Solvent	Temp (°C)	Yield (%) ^b^
1	Cu_2_O (20)	-	DMF	120	36
2	Cu_2_O (20)	-	DMSO	120	29
3	Cu_2_O (20)	-	NMP	120	33
4	Cu_2_O (20)	-	DMSO/NMP (4:1)	120	39
5	Cu_2_O (20)	-	DMF/NMP (4:1)	120	50
6	Cu_2_O (20)	-	DMF/NMP (9:1)	120	65
7	Cu_2_O (20)	-	DMF/NMP (9:1)	150	58
8	Cu_2_O (20)	-	DMF/NMP (9:1)	100	37
9 ^c^	Cu_2_O (20)	BF_3_·OEt_2_ (20)	DMF/NMP (9:1)	120	88 (83) ^d^
10 ^e^	Cu_2_O (10)	BF_3_·OEt_2_ (40)	DMF/NMP (9:1)	120	90 (83) ^d^
11	CuI (20)	BF_3_·OEt_2_ (40)	DMF/NMP (9:1)	120	69
12	Cu_2_O (10)	AlCl_3_ (40)	DMF/NMP (9:1)	120	48
13	Cu_2_O (10)	TiCl_4_ (40)	DMF/NMP (9:1)	120	37
14	Cu_2_O (10)	FeCl_3_ (40)	DMF/NMP (9:1)	120	trace
15	-	BF_3_·OEt_2_ (40)	DMF/NMP (9:1)	120	11

^a^ Reaction conditions: **1a** (0.2 mmol, 1.0 equiv), Cu (10–20 mol%), Lewis acid (0–40 mol%), solvent (2.0 mL), indicated temperature (°C), under N_2_ for 24 h. ^b 1^H NMR yield based on internal standard mesitylene; ^c^ 3.0 mL solvent was used; ^d^ isolated yield; ^e^ 36 h.

**Table 2:** Reaction scope ^a^.

	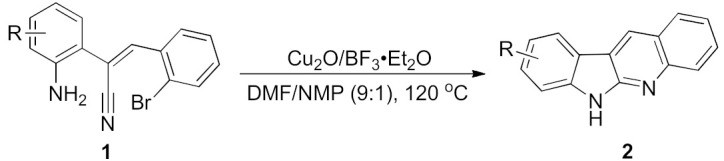			
Entry	1	2	Condition	Yield (%) ^b^
1	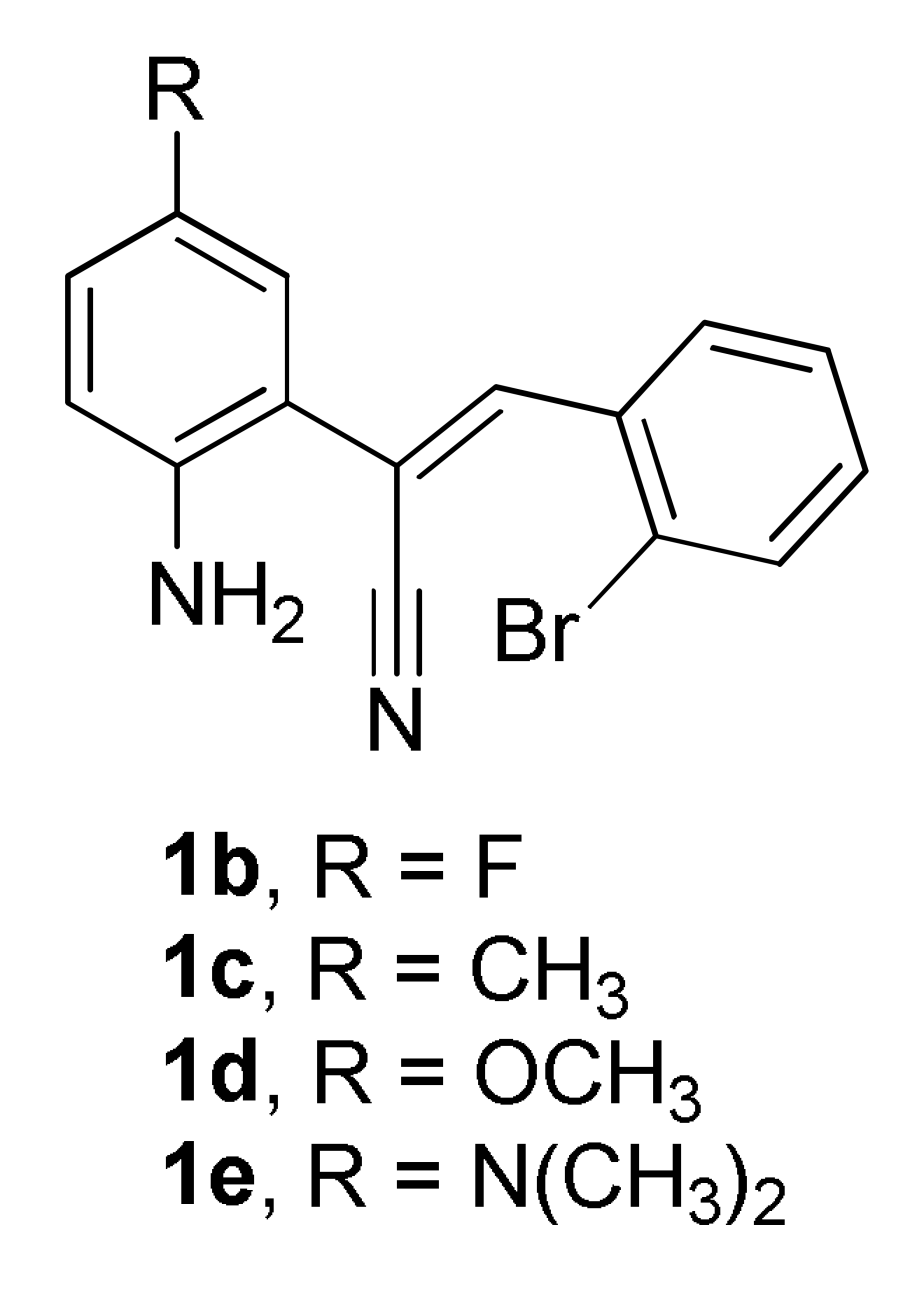	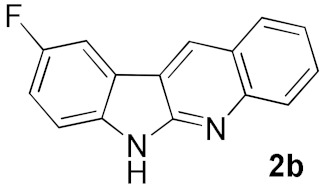	B	41
2		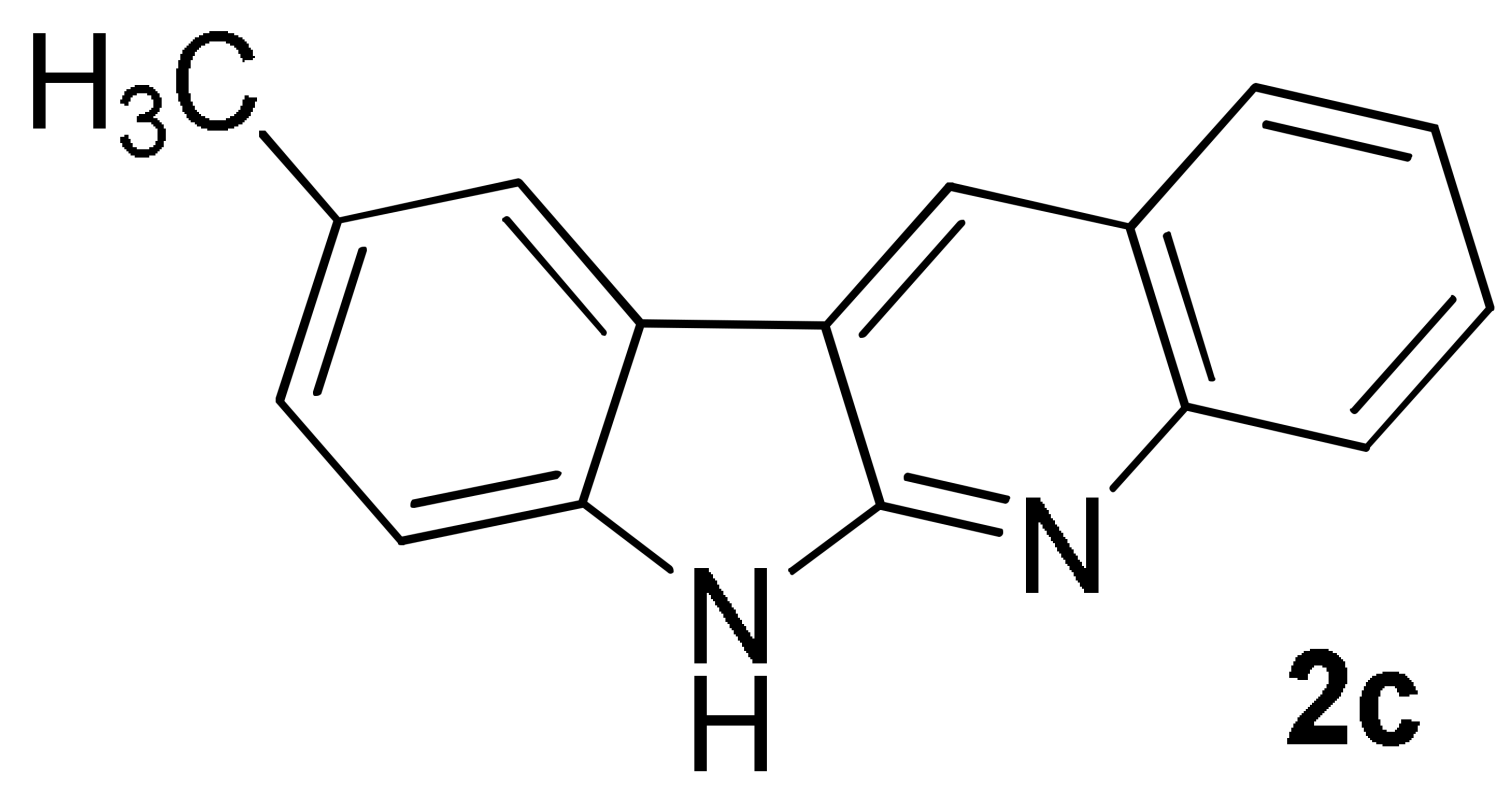	A	68
3		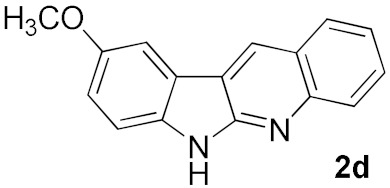	A	76
4		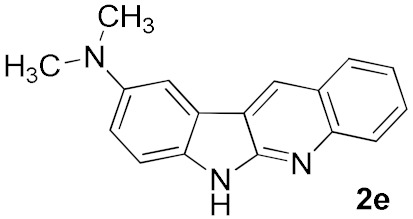	A	45
5	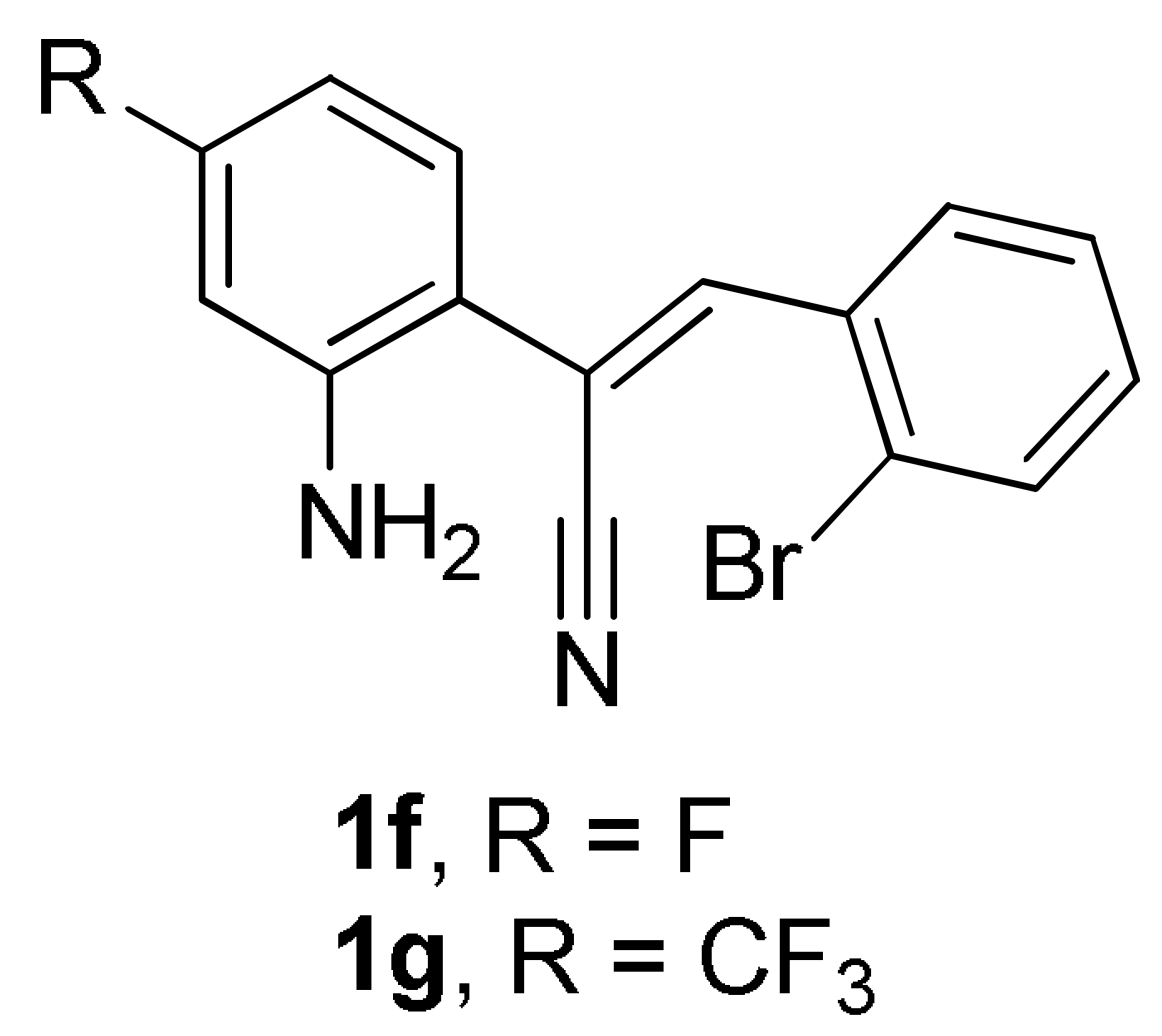	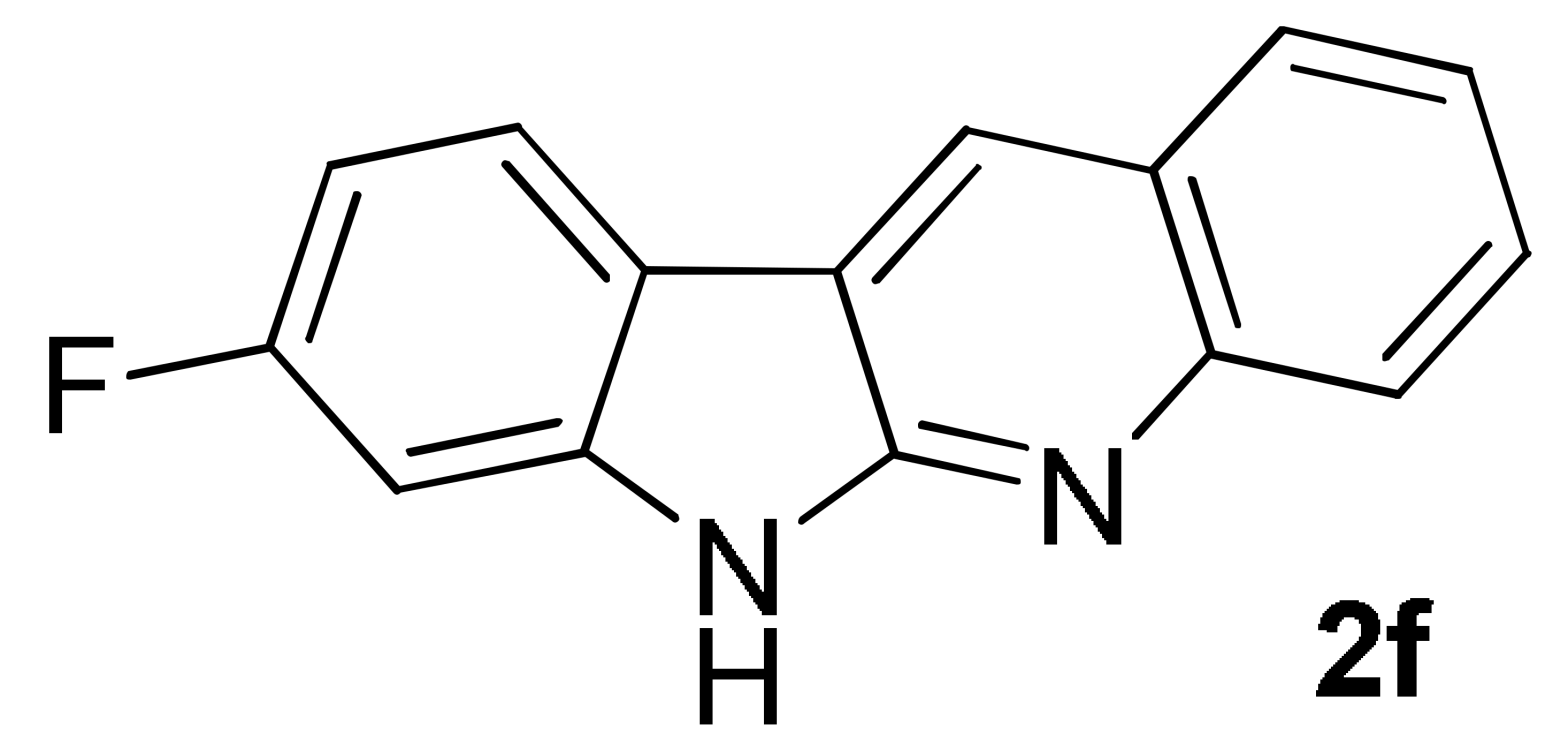	B	53
6		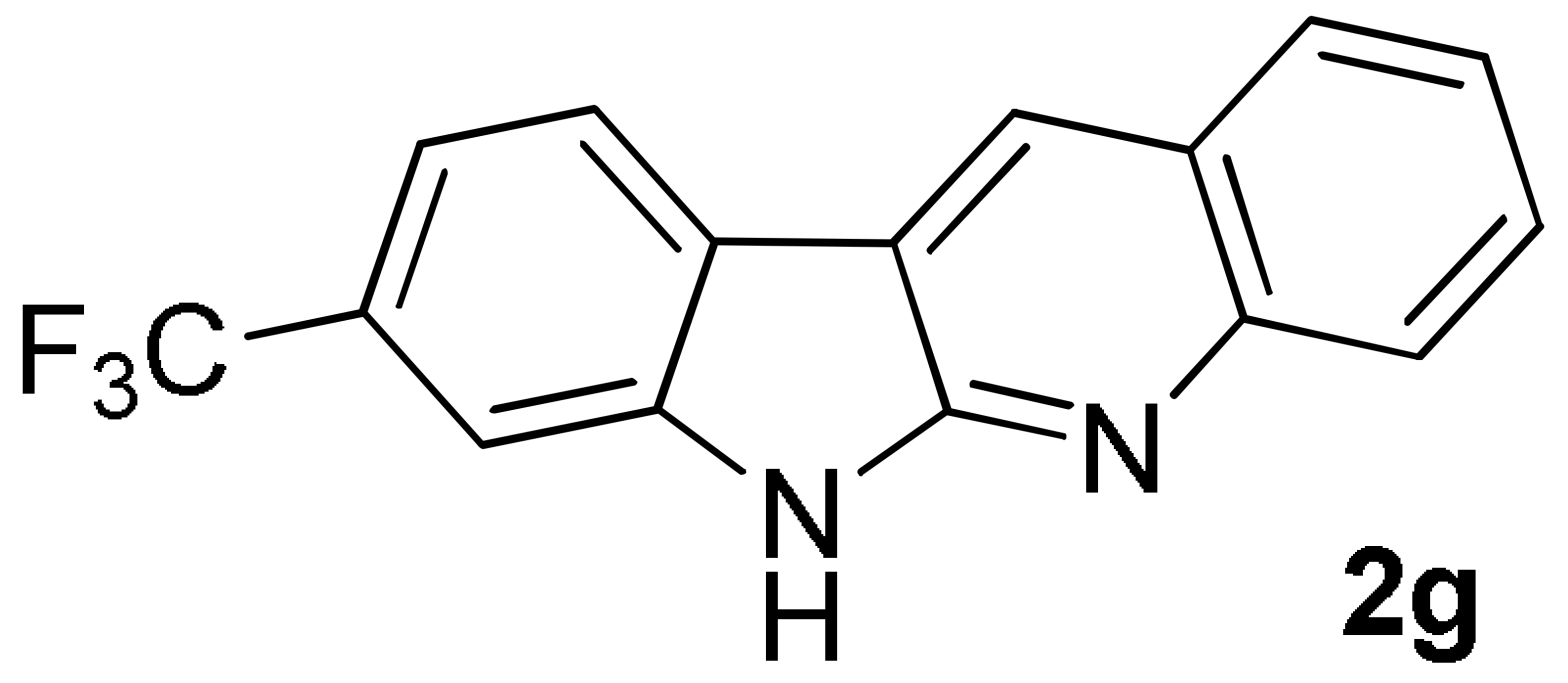	B	47
7	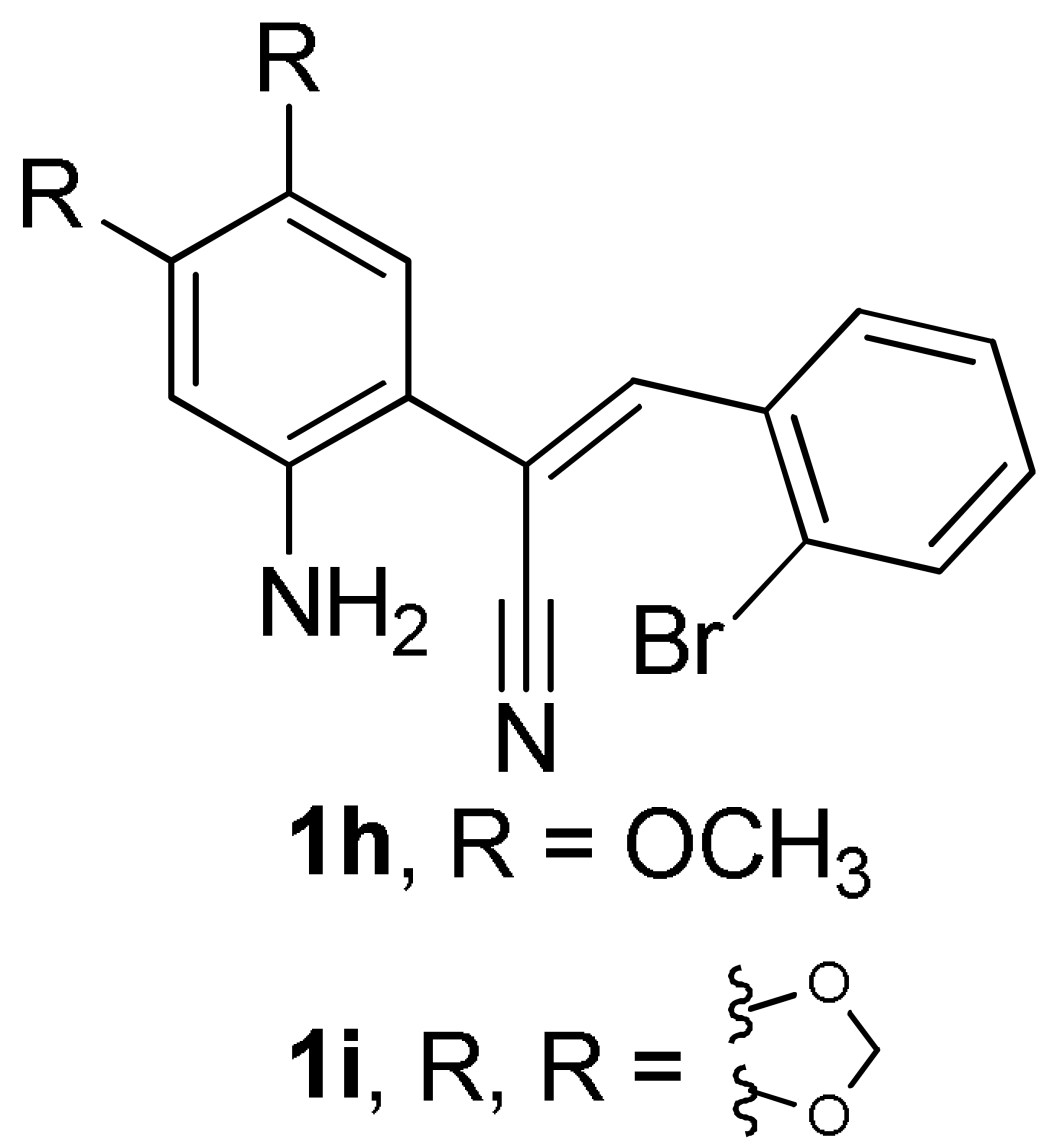	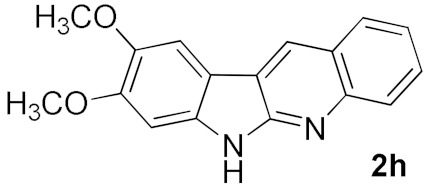	A	71
8		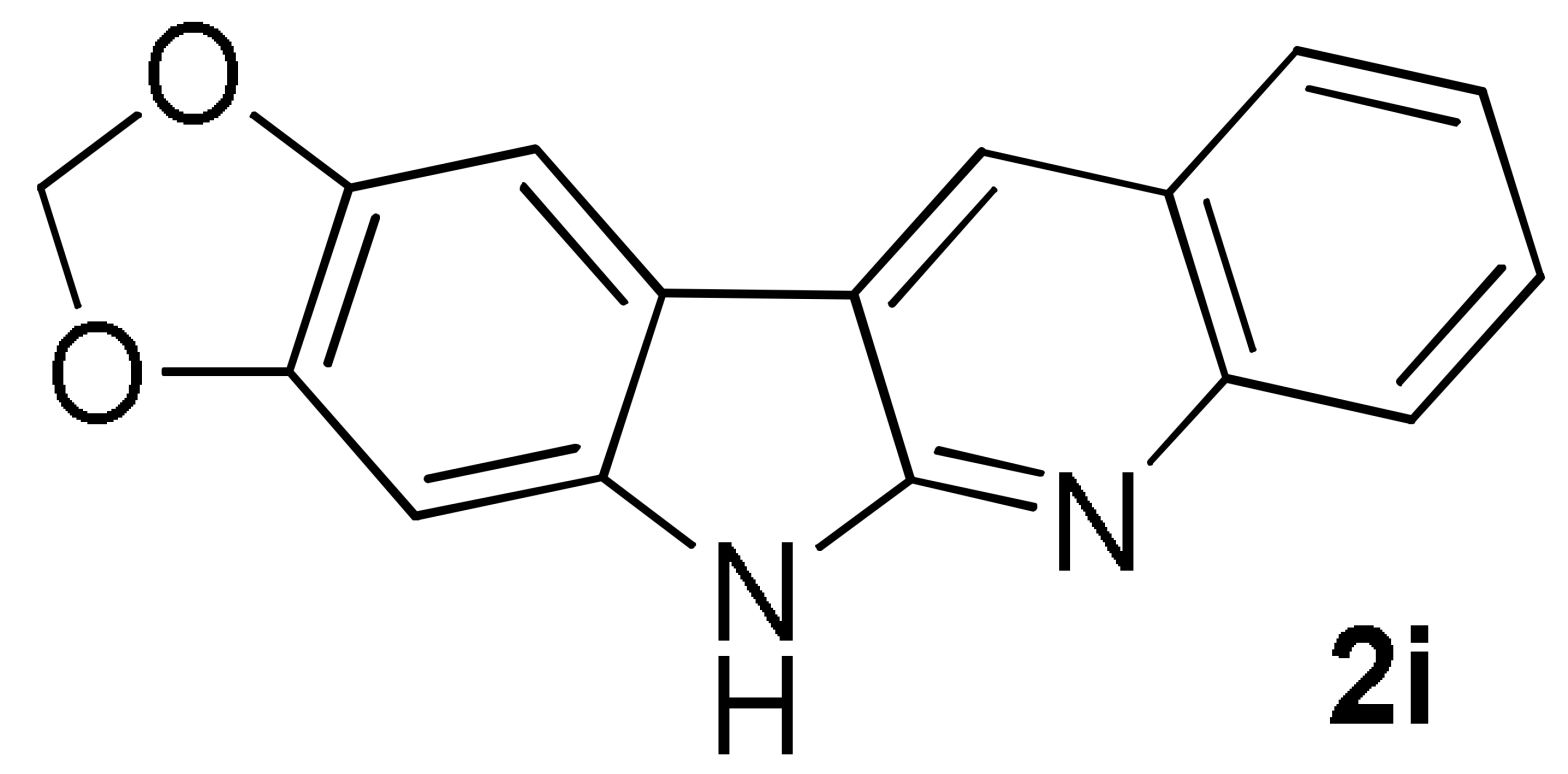	A	56

^a^ Condition A: **1** (0.4 mmol, 1.0 equiv), Cu_2_O (10 mol%), BF_3_·OEt_2_ (40 mol%), DMF/NMP (2.0 mL, 9:1), 120 °C, under N_2_ for 36 h. Condition B: **1** (0.4 mmol, 1.0 equiv), Cu_2_O (20 mol%), BF_3_·OEt_2_ 20 mol%), DMF/NMP (3.0 mL, 9:1), 120 °C, under N_2_ for 24 h. ^b^ Isolated yield.

**Table 3:** Reaction scope ^a^.

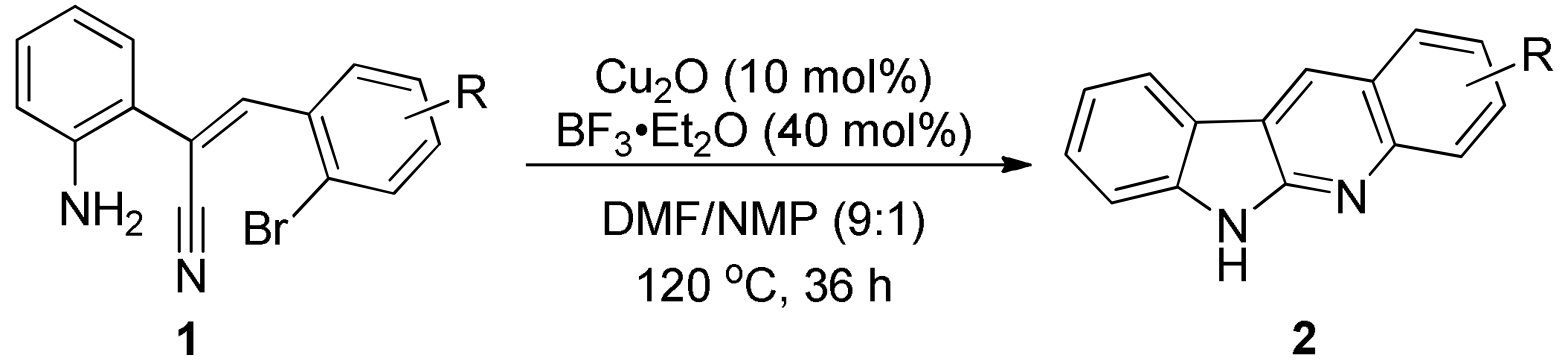			
**Entry**	**1**	**2**	**Yield (%) ^b^**
1	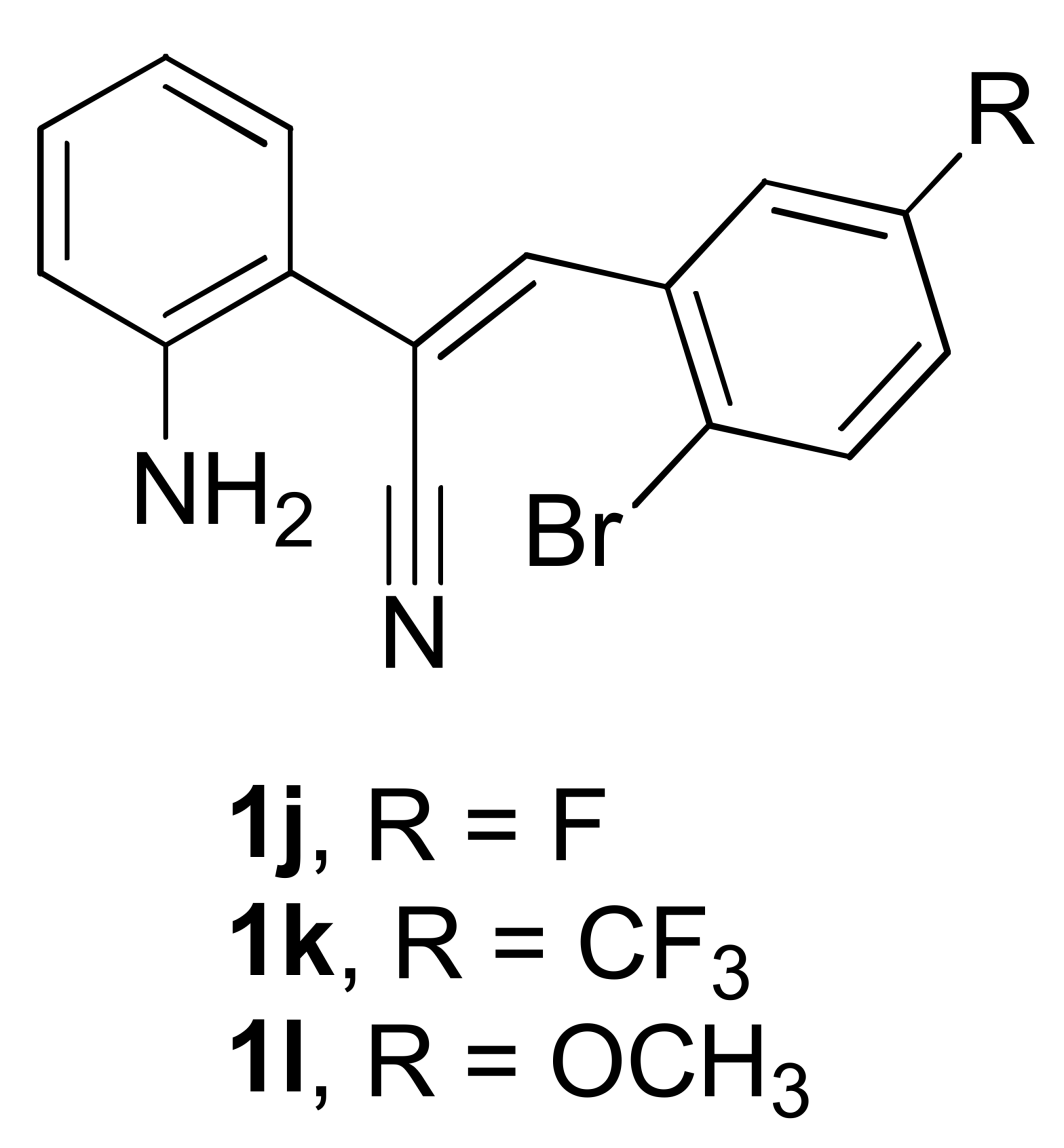	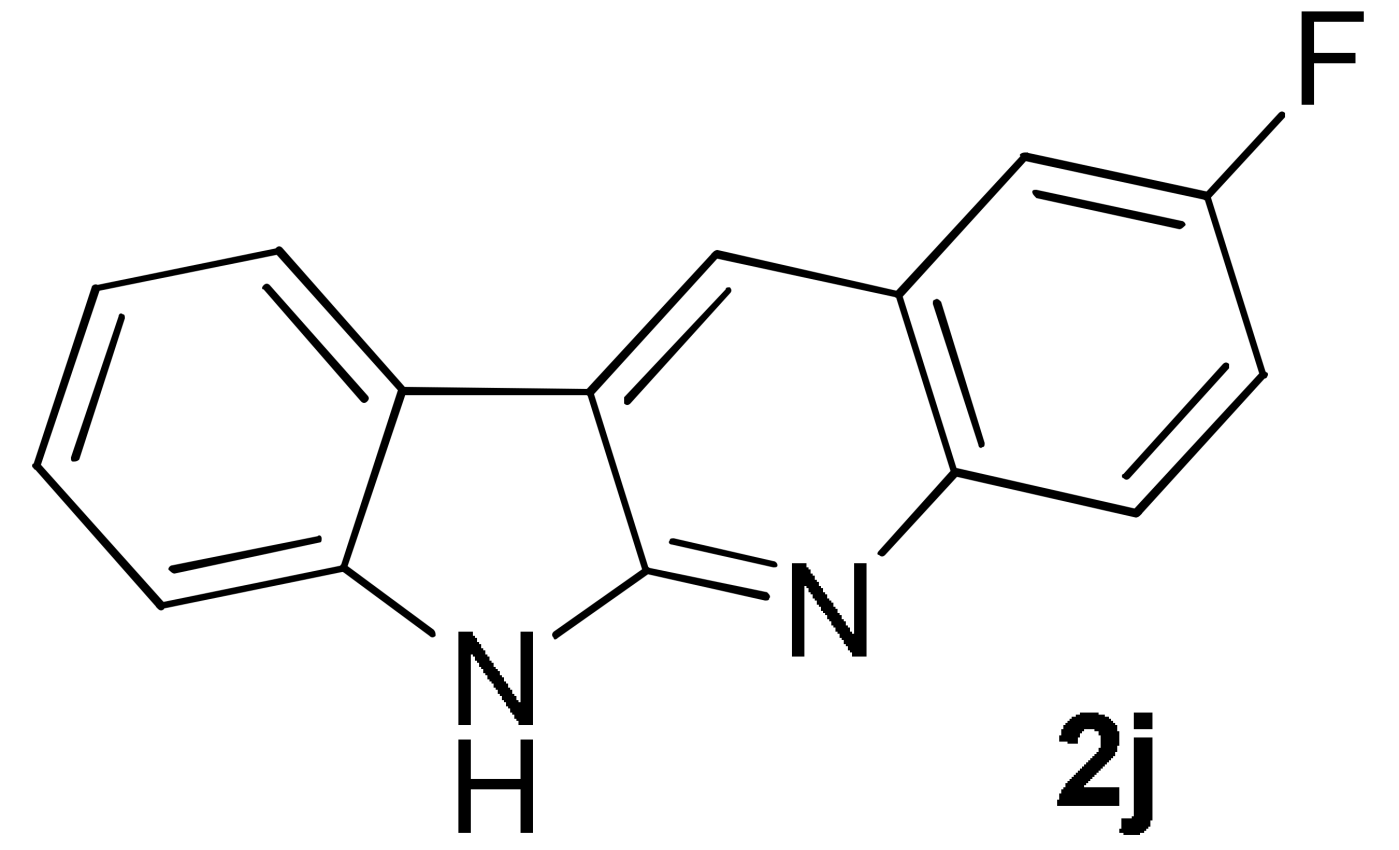	79
2		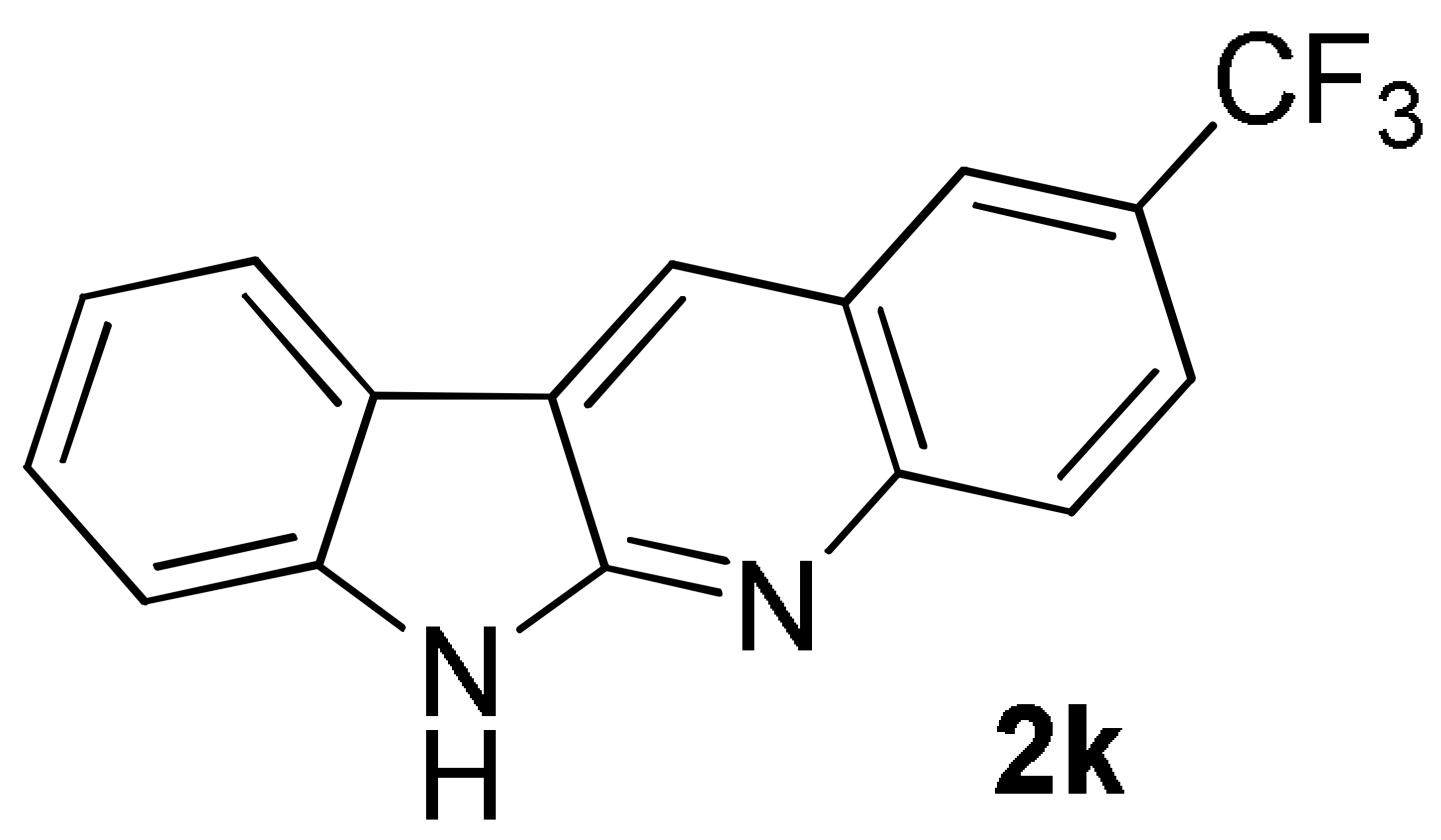	57
		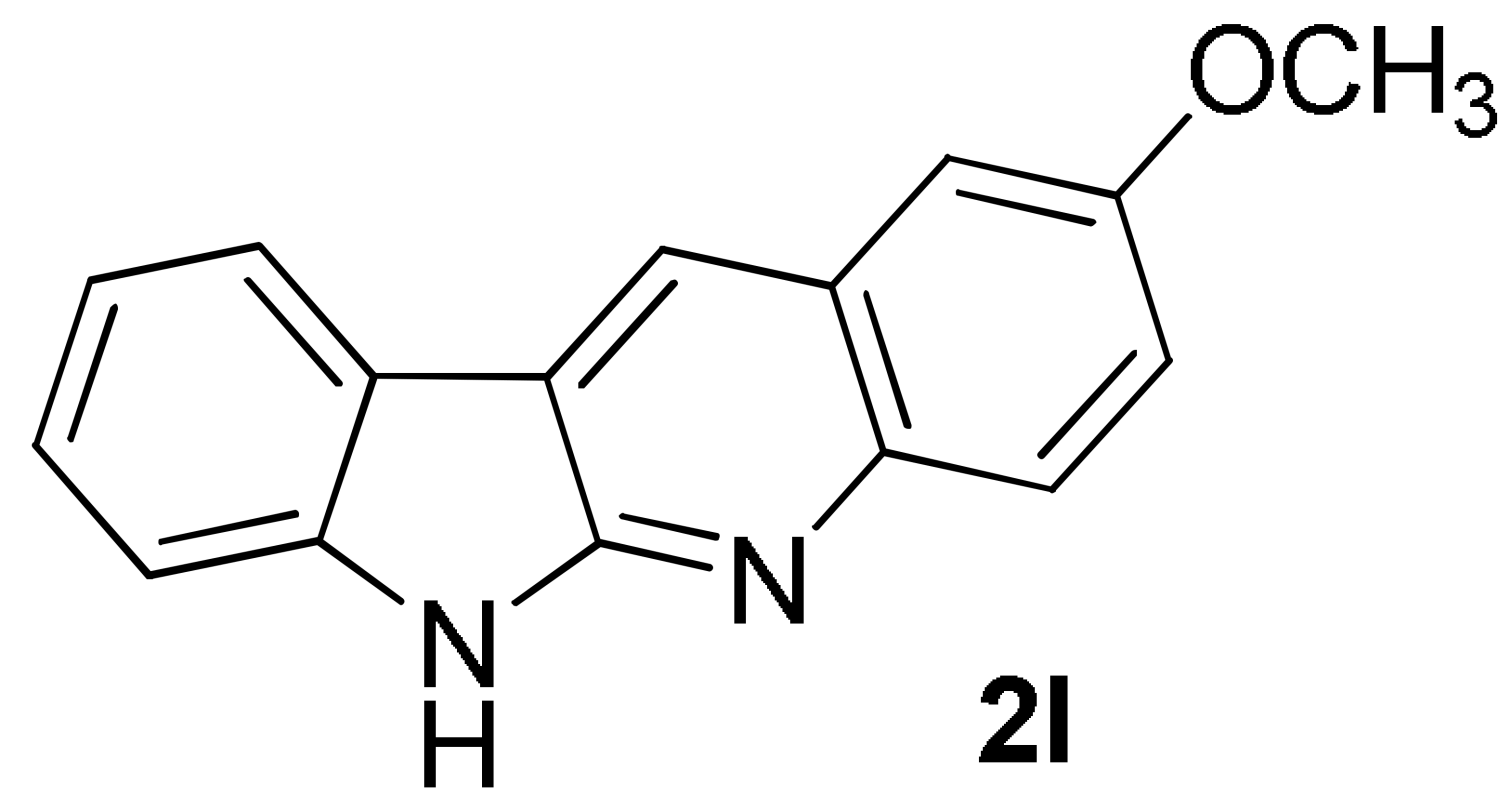	45
4	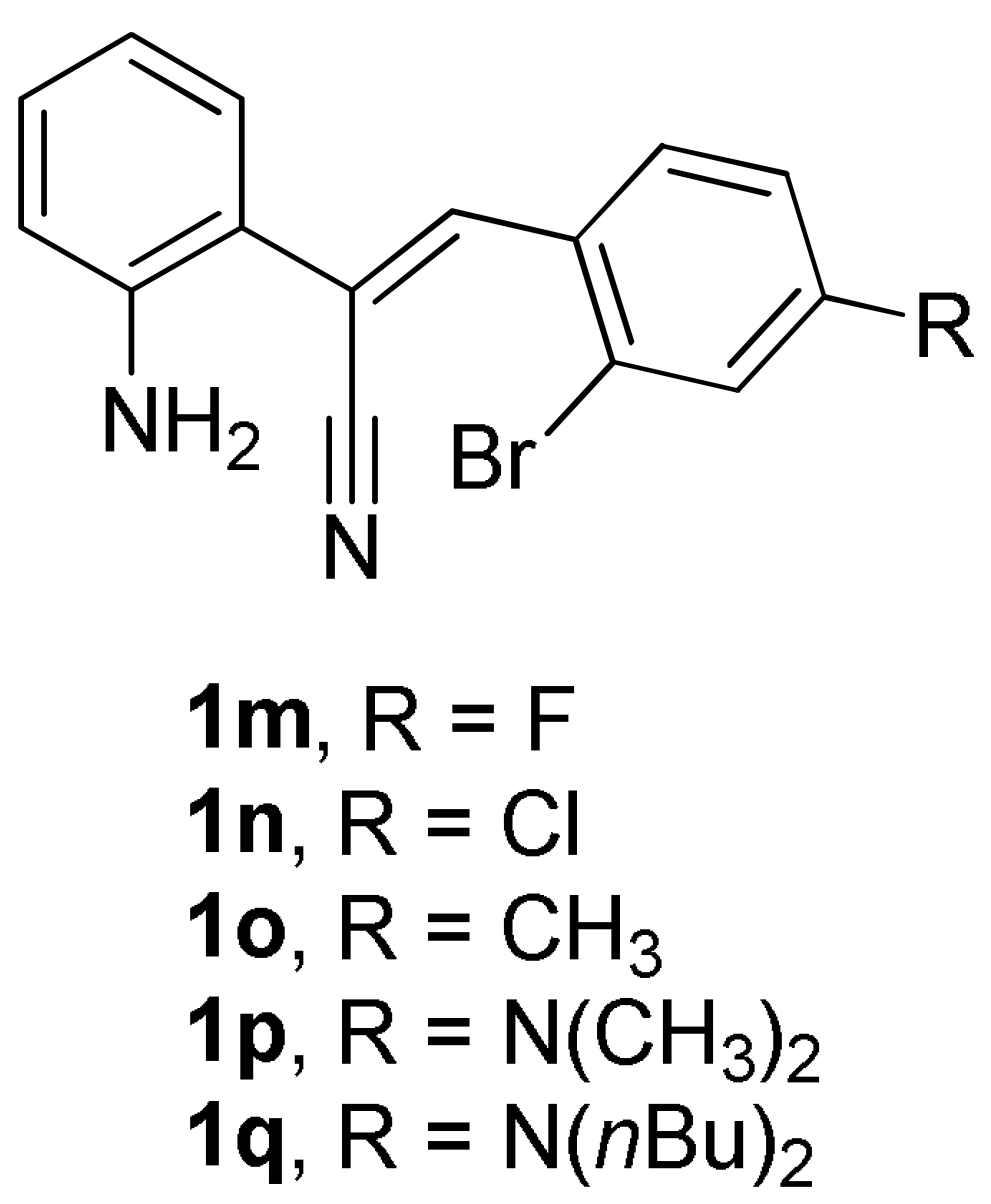	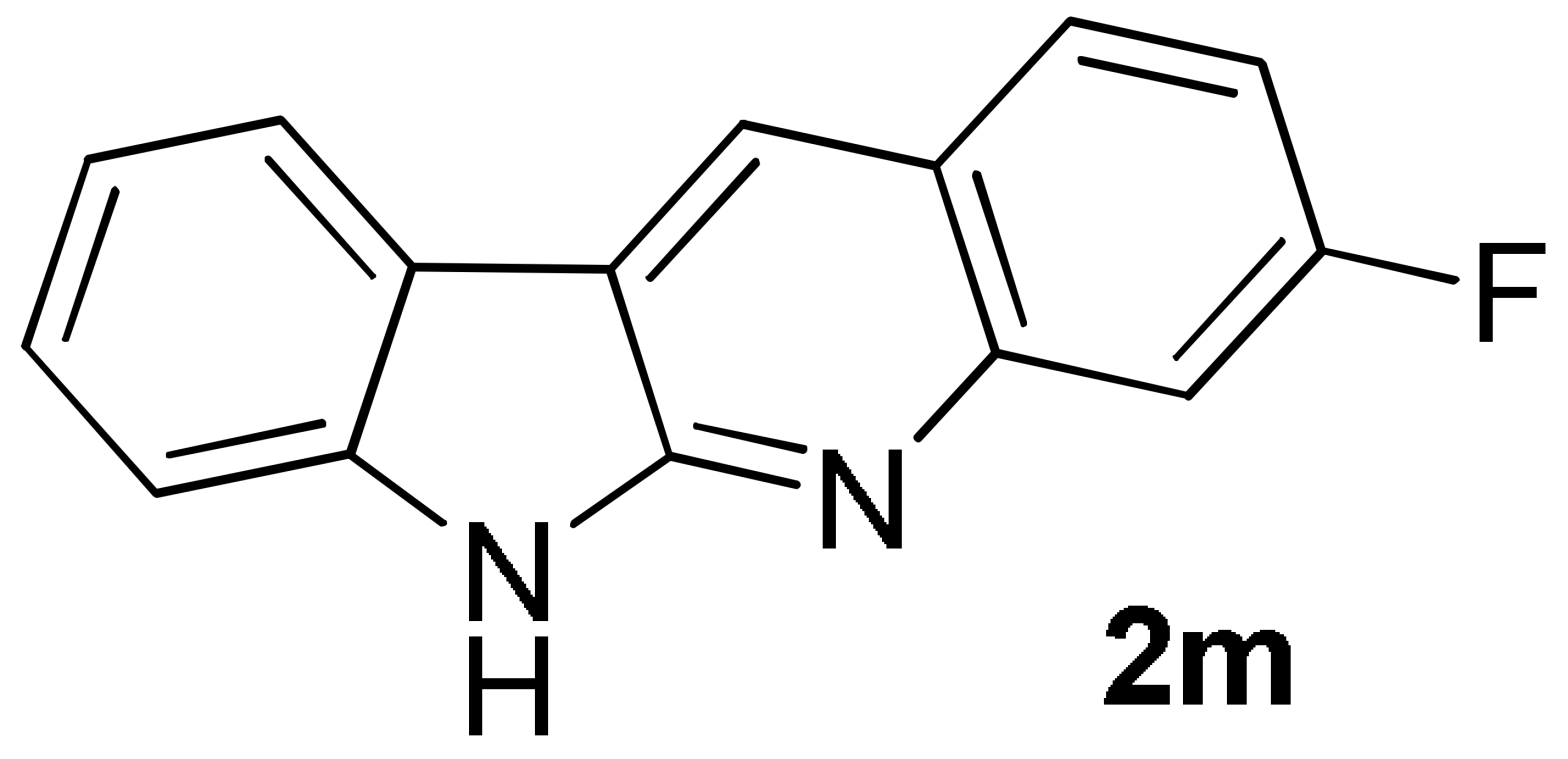	75
5		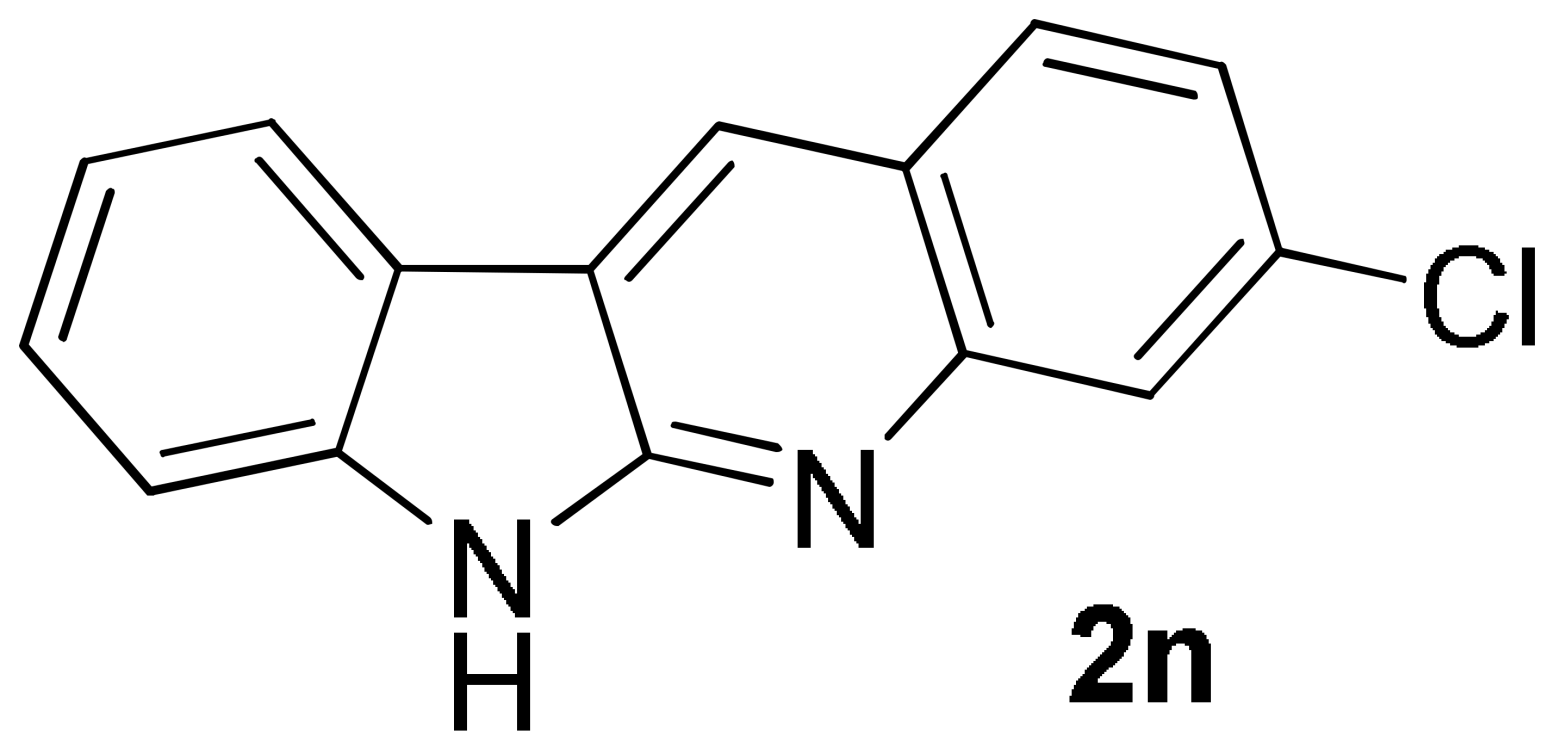	73
6		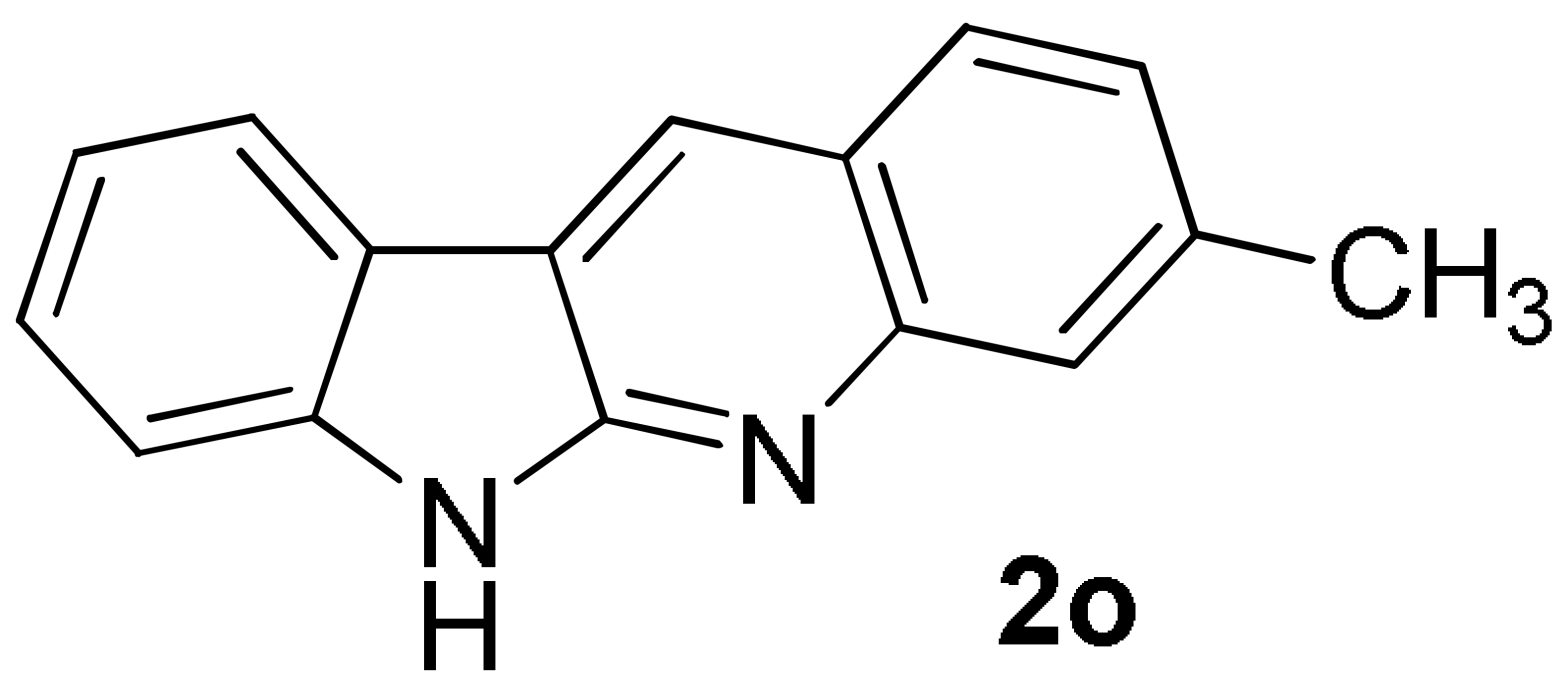	69
7		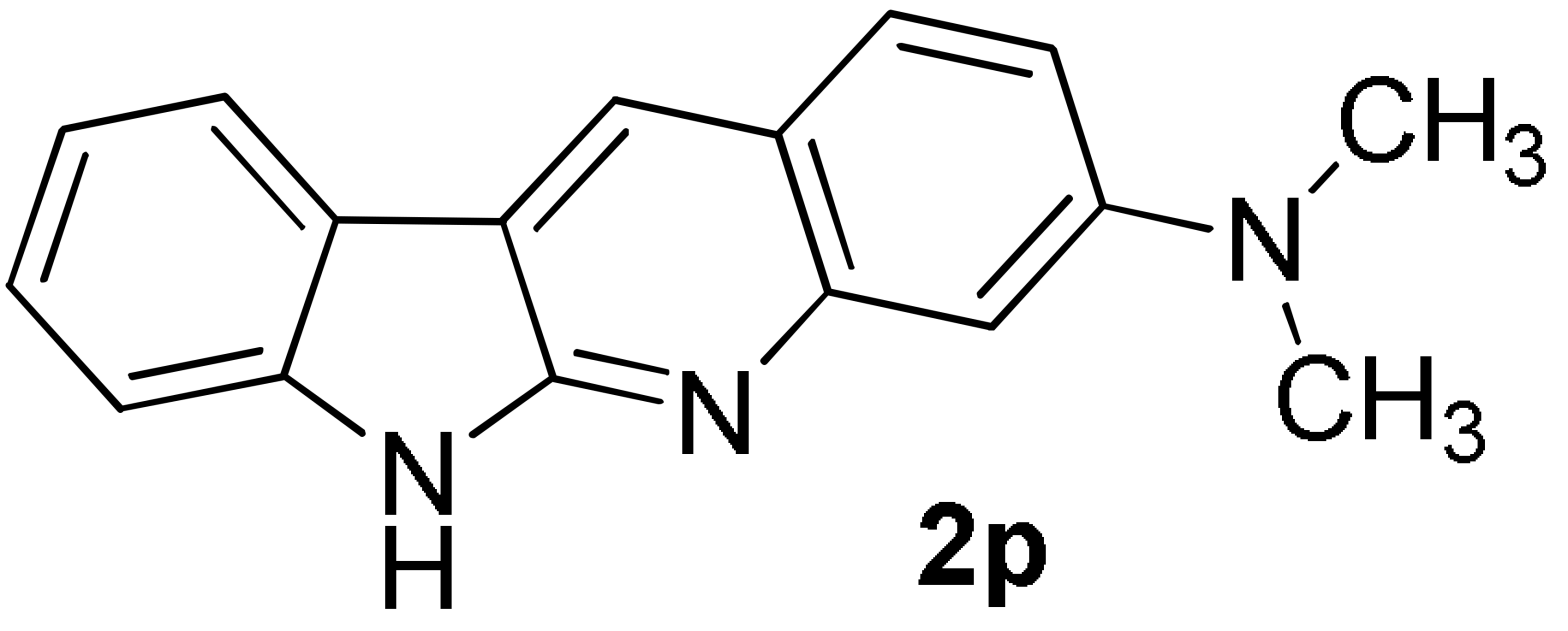	51
8		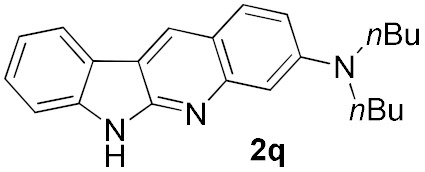	32
9	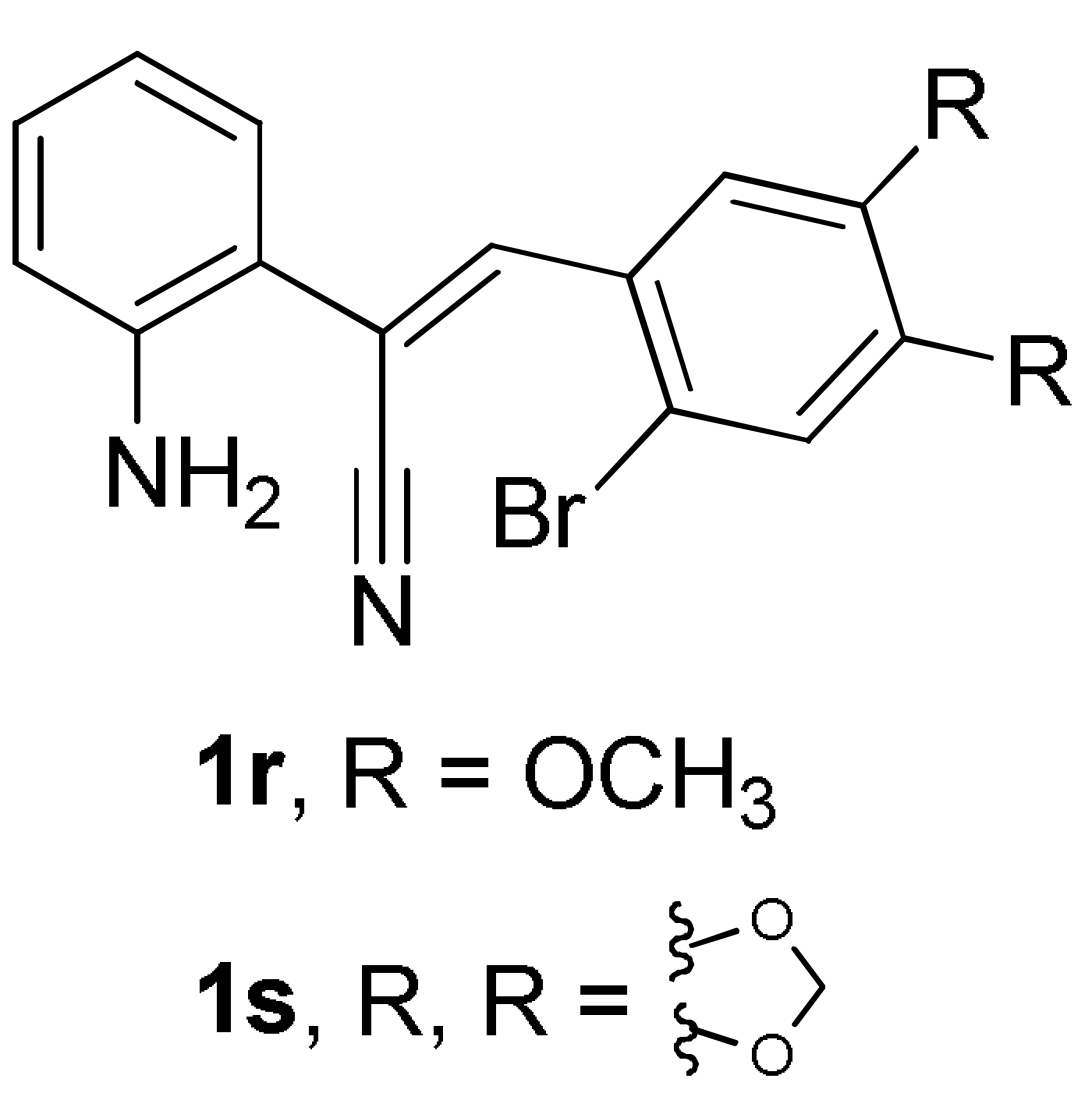	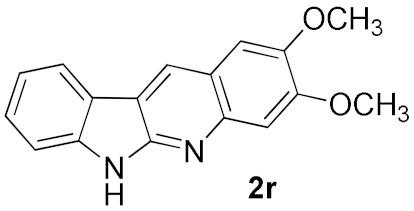	62
10		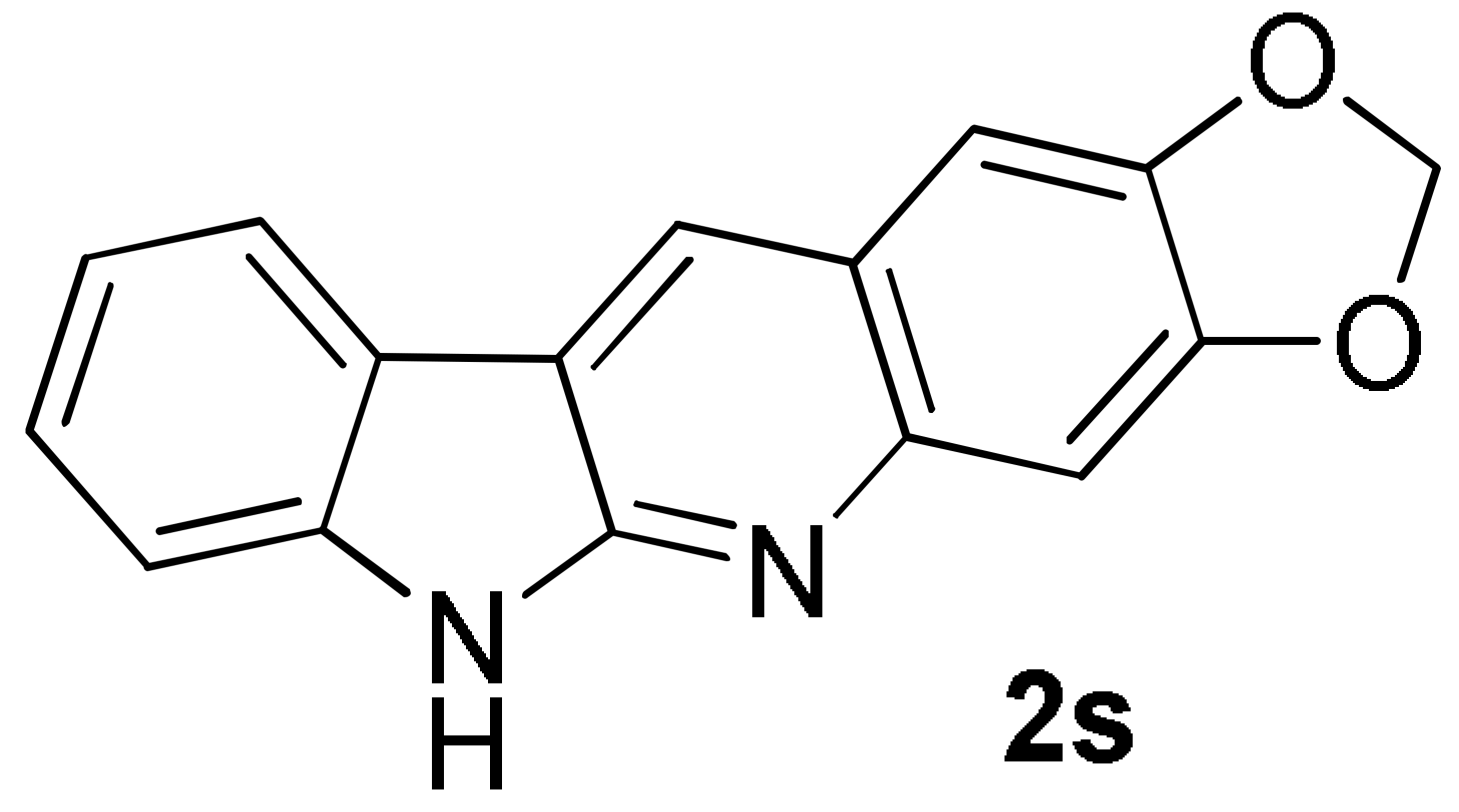	57
11	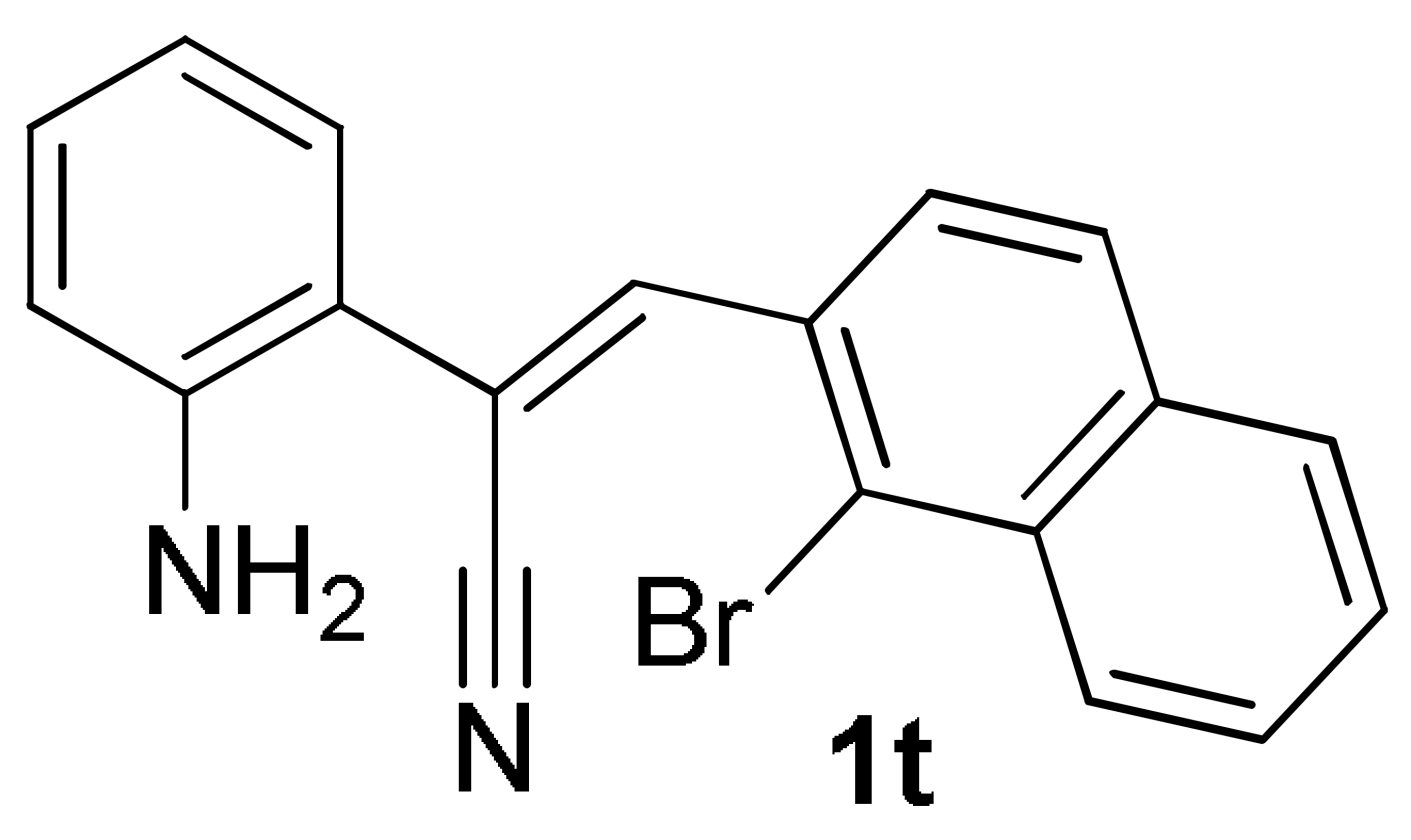	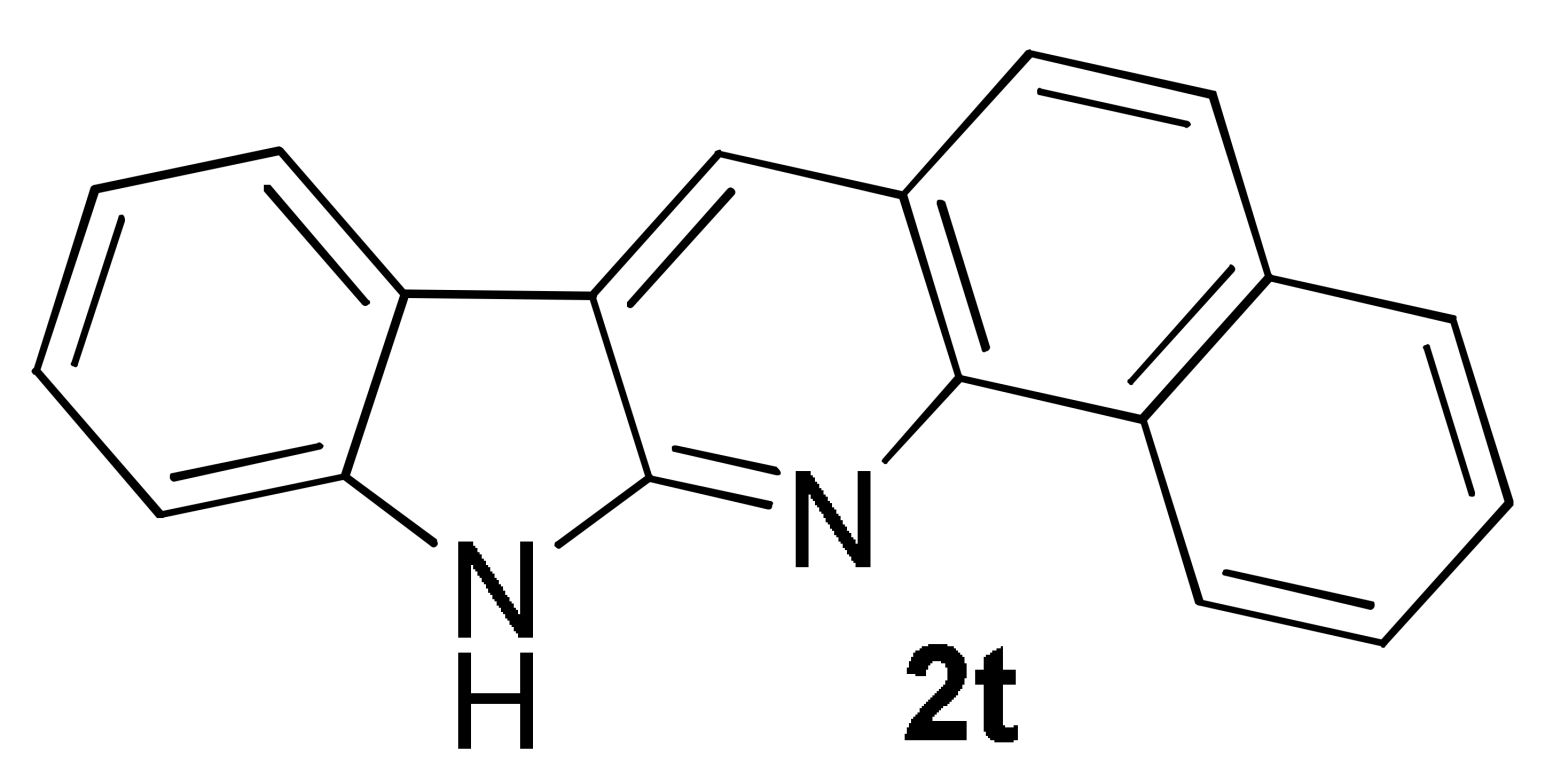	49

^a^ Reaction condition: **1** (0.4 mmol, 1.0 equiv), Cu_2_O (10 mol%), BF_3_·OEt_2_ (40 mol%), DMF/NMP (2.0 mL, 9:1), 120 °C, under N_2_ for 36 h. ^b^ Isolated yield.

## Supplementary Materials

The following are available online, experimental procedures for the synthesis of substrates (**1**) and products (**2**). Table S1–S3: optimization study.
