# Supplementary material for: Copper-Catalyzed Dual Cyclization for the Synthesis of Quinindolines
Source: Molecules. 2020 Nov 13;25(22):5303. doi: 10.3390/molecules25225303 (PMC7696892; doi:10.3390/molecules25225303)
Supplement: Supplementary file 1 [file molecules-25-05303-s001.pdf]

Supporting Information

Copper-Catalyzed Dual Cyclization for the Synthesis of Quinindolines

Hung-Kai Wang [1], Yu-Lun Ciou [1], Gangaram Pallikonda [1], Hsyueh-Liang Wu [2,]*, Haw-Lih Su [3] and Jen-Chieh Hsieh [1,]*

[1] Department of Chemistry, Tamkang University, New Taipei City, 25137, Taiwan (R.O.C.)
[2] Department of Chemistry, National Taiwan Normal University, Taipei, 11677, Taiwan (R.O.C.)
[3] Central Laboratories Unit, Qatar University, Doha, Qatar
[1,]*Correspondence: jchsieh@mail.tku.edu.tw; Tel.: +886-2-2621-5656 ext 2545.
[2,]*Correspondence: hlw@ntnu.edu.tw; Tel.: +886-2-7749-6142.

Table of Contents **Page No**

**General Information**	**S2**
**General procedure for the synthesis of quinindolines 2**	**S2**
**General procedure for the synthesis of substrate 1**	**S3**
**Optimization study**	**S3–S4**
**Reference**	**S5**

General information:

All reagents were purchased from Sigma-Aldrich, Alfa-Aesar, TCI and Fisher-Acros, which were used without further purification unless otherwise noted. All manipulations of oxygen- and moisture-sensitive materials were conducted with a standard Schlenk technique or in the glove box. Flash column chromatography was performed using silica gel (230-400 mesh). Analytical thin layer chromatography (TLC) was performed on 60 F_{254} (0.25 mm) plates and visualization was accomplished with UV light (254 and 354 nm) and/or an aqueous alkaline $KMnO_4$ solution followed by heating. Proton and carbon nuclear magnetic resonance spectra (^{1}H NMR and ^{13}C NMR) were recorded on Bruker 300 or Bruker 600 spectrometer with Me_4Si or solvent resonance as the internal standard (^{1}H NMR, Me_4Si at 0 ppm, $CDCl_3$ at 7.26 ppm, d_6-DMSO at 2.49 ppm; ^{13}C NMR, Me_4Si at 0 ppm, $CDCl_3$ at 77.0 ppm, d_6-DMSO at 39.7 ppm). ^{1}H NMR data are reported as follows: chemical shift, multiplicity (s = singlet, d = doublet, t = triplet, q = quartet, quint = quintet, sext = sextet, sept = septet, br = broad, m = multiplet), coupling constants (Hz), and integration. IR spectral data were recorded on a Bruker TENSOR 37 spectrometer. Melting points (mp) were determined using a SRS OptiMelt MPA100. GC-MS data were obtained from the HP 5890 Series II GC/HP 5972 GC MASS Spectrometer System. High Resolution Mass spectral data were obtained from MAT-95XL HRMS by using EI method. X-ray data was obtained from Bruker APEX DUO.

General procedure (A) for the synthesis of quinindolines 2:

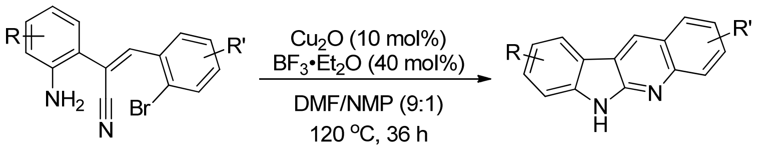

1 2

Addition of all reagents was conducted in the glove box. A screw-capped vial (20-mL) was added substrate **s** (0.4 mmol, 1.0 equiv), Cu_2O (5.7 mg, 0.04 mmol, 10 mol%), and $BF_3{\cdot}OEt_2$ (22.7 mg, 0.16 mmol, 40 mol%) in DMF/NMP (1.8 mL/0.2 mL) cosolvents. The vial was then removed from the glove box, and allowed to stir at 120 °C for 36 h, cooled and diluted with ethyl acetate, then filtered through a thin celite pad to remove the precipitate, concentrated *in vacuo*. The residue was purified through a column chromatography by using hexane and ethyl acetate as eluent to afford the desired products.

General procedure (B) for the synthesis of quinindolines 2:

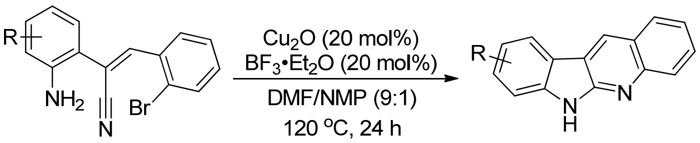

1 2

Addition of all reagents was conducted in the glove box. A screw-capped vial (20-mL) was added substrate **s** (0.4 mmol, 1.0 equiv), Cu_2O (11.3 mg, 0.08 mmol, 20 mol%), and $BF_3{\cdot}OEt_2$ (11.4 mg, 0.08 mmol, 20 mol%) in DMF/NMP (2.7 mL/0.3 mL) cosolvents. The vial was then removed from the glove box, and allowed to stir at 120 °C for 24 h, cooled and diluted with ethyl acetate, then filtered through a thin celite pad to remove the precipitate, concentrated *in vacuo*. The residue was purified through a column chromatography by using hexane and ethyl acetate as eluent to afford the desired products.

All structures were characterized by the HRMS, ^{1}H NMR and ^{13}C NMR spectra. Spectral data, melting point, IR data, HRMS data as well as the copies of ^{1}H NMR and ^{13}C NMR spectra for all compounds are recorded in reference 1, please see the reference for the detail.

General procedure for the synthesis of substrate 1[1]:

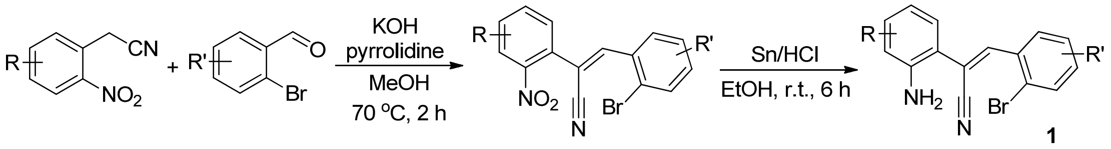

A solution of 2-(2-nitroaryl)acetonitrile (5 mmol, 1.0 equiv), 2-bromobenzaldehyde (5.5 mmol, 1.1 equiv) and pyrrolidine (7.0 mg, 0.1 mmol, 2 mol %) in MeOH (7.5 mL) was kept stirring at ambient temperature for 10 min. The reaction was added KOH solution (5% $KOH_{(aq)}$, 0.32 mL) and then kept stirring at 70 °C for 2 h. The crude reaction mixture was diluted with ethyl acetate, extracted by aqueous $NaHCO_3$. The combined organic layer was collected, dried over the $MgSO_4$ and concentrated *in vacuo*. The residue was washed with methanol to give the desired compound, which can be directly used for the next step.

To a round bottom flask (50-mL) were added the above NO_2-compound (6 mmol, 1.0 equiv), Sn powder (7.2 g, 60 mmol, 10 equiv) and EtOH (15 mL). The mixture was kept stirring at ambient temperature, and then the conc. HCl (12 M, 18 mL) was slowly injected to the solution mixture. After complete the injection, the solution was kept stirring at ambient temperature for another 6 h. The crude reaction mixture was diluted with ethyl acetate, extracted by aqueous $NaHCO_3$. The combined organic layer was collected, dried over the $MgSO_4$ and concentrated *in vacuo*. The residue was purified through flash column chromatography by using hexane and ethyl acetate as eluent to give substrate **1**.

Table S1. Optimization study*[a]*

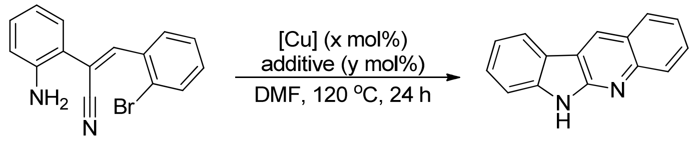

entry	[Cu] (x)	additive (y)	yield (%)[b]	entry	[Cu] (x)	additive (y)	yield (%)[b]
1	CuI (20)	-	23	14	Cu_2O (20)	$AlMe_3$ (20)	51
2	CuBr (20)	-	14	15	Cu_2O (20)	$TiCl_4$ (20)	51
3	CuCl (20)	-	13	16	Cu_2O (20)	$Ag(OTf)_2$ (20)	38
4	$Cu(OAc)_2$ (20)	-	14	17	Cu_2O (20)	$MgBr_2$ (20)	47
5	$CuCl_2$ (20)	-	21	18	Cu_2O (20)	$LaCl_3$ (20)	39
6	$Cu(TFA)_2$ (20)	-	23	19	Cu_2O (20)	$BF_3·OEt_2$ (20)	83
7	$Cu(OTf)_2$ (20)	-	23	20	CuI (20)	$BF_3·OEt_2$ (20)	64
8	Cu_2O (20)	-	36	21	$CuCl_2$ (20)	$BF_3·OEt_2$ (20)	67
9	Cu_2O (10)	-	20	22	Cu_2O (10)	$BF_3·OEt_2$ (30)	63
10	Cu_2O (20)	-	36	23	Cu_2O (10)	$BF_3·OEt_2$ (40)	83
11	Cu_2O (20)	$ZnCl_2$ (20)	40	24	Cu_2O (10)	$BF_3·OEt_2$ (50)	71
12	Cu_2O (20)	$ZnBr_2$ (20)	38	25[c]	Cu_2O (10)	$BF_3·OEt_2$ (40)	85
13	Cu_2O (20)	$AlCl_3$ (20)	69	26[c]	CuI (20)	$BF_3·OEt_2$ (40)	69

[a]Reaction conditions: **1a** (0.2 mmol, 1.0 equiv), [Cu] (10–40 mol%), additive (0–50 mol%) in 2.0 mL DMF at 120 °C, under N_2 for 24 h. [b] ^1H NMR yield based on internal standard mesitylene. [c] 36 h.

Table S2. Optimization study*a*

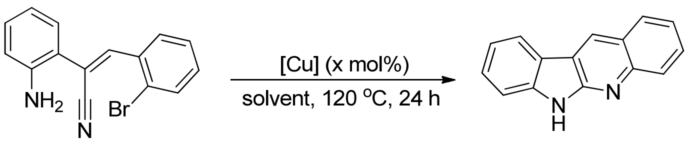

entry	[Cu] (x)	solvent	yield (%)*b*	entry	[Cu] (x)	solvent	yield (%)*b*
1	CuI (20)	*t*BuOH	9	10	CuI (20)	NMP	18
2	CuI (20)	*i*PrOH	7	11	Cu_2O (20)	DMF	36
3	CuI (20)	1,4-dioxane	8	12	Cu_2O (20)	DMSO	29
4	CuI (20)	toluene	trace	13	Cu_2O (20)	DMA	11
5	CuI (20)	o-xylene	trace	14	Cu_2O (20)	NMP	33
6	CuI (20)	THF	trace	15	Cu_2O (20)	DMF/THF (4:1)	46
7	CuI (20)	DMF	23	16	Cu_2O (20)	DMF/NMP (4:1)	50
8	CuI (20)	DMSO	21	17	Cu_2O (20)	DMSO/NMP (4:1)	39
9	CuI (20)	DMA	10	18	Cu_2O (20)	DMF/NMP (9:1)	65

[a]Reaction conditions: **1a (**0.2 mmol, 1.0 equiv), [Cu] **(**20 mol%) in 2.0 mL solvent at 120 °C for 24 h. [b] ^{1}H NMR yield based on internal standard mesitylene.

Table S3. Optimization study*a*

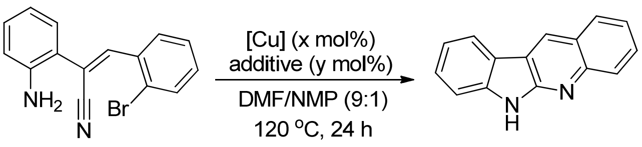

entry	[Cu] (x)	additive (y)	yield (%)*b*	entry	[Cu] (x)	additive (y)	yield (%)*b*
1	CuI (20)	-	23	11	Cu_2O (20)	$BF_3·OEt_2$ (10)	63
2	CuI (20)	$BF_3·OEt_2$ (40)	69	12	Cu_2O (20)	$AlCl_3$ (20)	76
3	$Cu(OTf)_2$ (20)	$BF_3·OEt_2$ (40)	71	13	Cu_2O (10)	$AlCl_3$ (40)	48
4	$Cu(TFA)_2$ (20)	$BF_3·OEt_2$ (40)	67	14	Cu_2O (20)	$TiCl_4$ (20)	53
5	Cu_2O (10)	$BF_3·OEt_2$ (40)	81	15	Cu_2O (10)	$TiCl_4$ (40)	37
6[c]	Cu_2O (10)	$BF_3·OEt_2$ (40)	90	16	Cu_2O (10)	$FeCl_3$ (40)	trace
7[c]	Cu_2O (5)	$BF_3·OEt_2$ (40)	55	17	Cu_2O (10)	$ZnCl_2$ (40)	29
8	Cu_2O (20)	$BF_3·OEt_2$ (30)	84	18	Cu_2O (10)	$CuCl_2$ (40)	82
9	Cu_2O (20)	$BF_3·OEt_2$ (20)	83	19	Cu_2O (10)	HCl (40)	trace
10[d]	Cu_2O (20)	$BF_3·OEt_2$ (20)	88	20	Cu_2O (10)	*p*TSA (40)	0

[a]Reaction conditions: **1a (**0.2 mmol, 1.0 equiv), [Cu] **(**5–20 mol%), additive (0–40 mol%) in 2.0 mL solvent at 120 °C for 24 h. [b] ^{1}H NMR yield based on internal standard mesitylene. [c] 36 h. [d] 3.0 mL solvent

References

(1) Yeh, L.-H.; Wang, H.-K.; Pallikonda, G.; Ciou, Y.-L.; Hsieh, J.-C. Palladium-Catalyzed Dual Annulation: A Method for the Synthesis of Norneocryptolepine. *Org. Lett.* **2019**, *21*, 1730–1734.
